# Translating spatial navigation evaluation from experimental to clinical settings: The virtual environments navigation assessment (VIENNA)

**DOI:** 10.3758/s13428-023-02134-0

**Published:** 2023-05-11

**Authors:** Sophia Rekers, Carsten Finke

**Affiliations:** 1 Department of Neurology, Charité - Universitätsmedizin Berlin, corporate member of Freie Universität Berlin and Humboldt-Universität zu Berlin, Charitéplatz 1, 10117 Berlin, Germany; 2 Berlin School of Mind and Brain, Humboldt-Universität zu Berlin, Berlin, Germany

**Keywords:** Spatial navigation, Topographical orientation, Route navigation, Neuropsychology, Virtual environments, Cognitive assessment, Aging

## Abstract

Spatial navigation abilities are frequently impaired in neurological disorders and they also decline with normal aging. Researchers and clinicians therefore need valid and easy-to-use spatial navigation assessment tools to study the impact of different neuropathologies and prevent relevant cognitive impairments from going undetected. However, current experimental paradigms rarely address which cognitive processes they recruit, often have resource-intensive setups, and usually require active navigation, e.g., using a joystick or keyboard, thus confounding cognitive performance with fine motor skills. Yet, for clinical feasibility, time-efficient paradigms are needed that are informative and easy to administer in participants with limited technical experience and diverging impairments. Here, we introduce the virtual environments navigation assessment (VIENNA), a virtual adaptation of a brief, standardized, and intuitive spatial navigation paradigm (https://osf.io/kp4c5/). VIENNA is designed to assess spatial navigation without episodic memory demands, requires no interface device, and takes about 16 min to complete. We evaluated VIENNA in 79 healthy middle-aged to older participants (50–85 years) and provide evidence for its feasibility and construct validity. Tests of visuospatial and executive functions, but not episodic memory or selective attention, were identified as cognitive correlates of VIENNA, even when controlling for participant age and overall cognitive performance. Furthermore, VIENNA scores correlated with subjective navigation ability and age, but not with depressiveness, cognitive complaints, or education. The straightforward administration of VIENNA allows for its integration into routine neuropsychological assessments and enables differentiated evaluation of spatial navigation performance in patients with motor impairments and episodic memory deficits.

**Supplementary Information:**

The online version contains supplementary material available at 10.3758/s13428-023-02134-0.

Spatial navigation impairments have been suggested as early diagnostic and functional markers in different neurological disorders [e.g., Alzheimer’s disease (Coughlan et al., 2018); Parkinson’s disease (Thurm et al., 2016); multiple sclerosis (Němá et al., 2021)]. There is a wealth of well-established experimental paradigms to assess spatial navigation. Many of the more common assessments focus on episodic memory and learning, and vary considerably in terms of the exact behavior and performance they measure [e.g., spatial reference memory in virtual arena tasks (Doeller et al., 2010), map encoding and active wayfinding in Sea Hero Quest (Coutrot et al., 2018), or route-learning (test suite by Wiener et al., 2020)]. In addition, the translation of navigation assessments to clinical evaluation is still pending, e.g., due to complex experimental setups and time- and resource-restrictions in clinical settings. Consequently, navigation abilities are frequently only approximated by qualitative behavioral descriptions. To objectify potential deficits, a navigation assessment should be easily applicable in older adults and should be inert to potential inexperience with technology and mild perceptual and motor impairments. To address these issues, we developed the brief, intuitive, and ecologically valid virtual environments navigation assessment (VIENNA), which is freely available for clinicians and researchers (https://osf.io/kp4c5/).

Spatial navigation relies on a complex interplay of different cognitive functions and cannot be captured using established neuropsychological paradigms on verbal and nonverbal memory, executive functions, attention, or working memory (Laczó et al., 2017). The cognitive components of a navigation assessment are strongly influenced by the objectives of the task and the availability of spatial representations. Navigation tasks can require participants to identify or memorize objects or landmarks (landmark knowledge), a location of a landmark (location knowledge), or a path to a certain goal (route or survey knowledge) (Claessen & van der Ham, 2017; Montello, 1998). In these tasks, the extent to which participants need to supplement online available spatial representations (i.e., currently available cues like one’s orientation on a route) with offline spatial representations (i.e., information on the route or environment which need to be recalled, like a memorized map) (Wolbers & Hegarty, 2010) affects the extent of episodic memory recruitment. In recent patient lesion studies, evidence for a partial dissociation between episodic memory requirements and navigation emerged with memory-impaired patients exhibiting intact navigation performance (Iggena et al., 2022; McAvan et al., 2022; Urgolites et al., 2016).

While spatial navigation and episodic memory share a network of regions, which are recruited by both functions, the retrosplenial cortex and other more posterior regions are particularly important for navigation (Ekstrom & Hill, 2023; Teghil et al., 2021). The relevance of a navigation assessment without an explicit episodic memory component becomes evident since many of these shared regions are also implicated during navigation in new and dynamic environments via functions with limited episodic memory involvement. These include the prefrontal and anterior cingulate cortex in the executive domain for route planning, processing deviations from the original route, and choosing and maintaining a goal while navigating (Patai & Spiers, 2021); the precuneus for maintaining and updating spatial information during online navigation (Jahn et al., 2012; Wolbers et al., 2008); and the retrosplenial cortex and parieto-occipital cortex for the processing of different spatial perspectives or reference frames (Mitchell et al., 2018; Vann et al., 2009) and imagining shifts in these reference frames—or perspective taking (Hegarty & Waller, 2004; Lambrey et al., 2012).

The recruited brain networks are also influenced by which perspective is processed during navigation. A distinction is made between egocentric and allocentric perspectives. The allocentric perspective refers to the relationship of environmental cues to one another, also called survey or top-down navigation, and is associated with the hippocampus interacting with a network of prefrontal, parietal, retrosplenial, and parahippocampal activation (Ekstrom et al., 2014). It has been shown that damage to the hippocampus can selectively affect allocentric spatial memory in contrast to egocentric memory representations (Finke et al., 2011). The egocentric perspective refers to the relationship of the navigator to environmental cues. It is particularly used for path or route-based navigation, also called sequential or response-based navigation (Ekstrom et al., 2018; Waller & Lippa, 2007). These egocentric navigation types have been associated with parietal activation but additionally with the striatum (Goodroe et al., 2018) and route navigation specifically with the caudate nucleus (Hartley et al., 2003).

The distinction between egocentric and allocentric navigation is particularly important when looking at the negative effect of aging on navigation performance, which is a robust finding that cannot be explained by a decline in episodic memory and does not affect all aspects of navigation uniformly (Lester et al., 2017; Moffat, 2009). Regarding basic processing of spatial information, distance perception seems to be intact in older compared to younger adults (Bian & Andersen, 2013), and minor changes in the perception of self-motion cues [speed (Lalonde-Parsi & Lamontagne, 2015) and direction (Warren et al., 1989)] have been identified. Looking at the effect of aging on specific navigation tasks, landmark knowledge is relatively spared, while the dynamic process of navigation is most affected by aging (van der Ham & Claessen, 2020). Importantly, allocentric, i.e., hippocampus-dependent navigation, appears to be more difficult for older adults, resulting in a greater reliance on egocentric and cue-based approaches associated with the caudate nucleus (Harris & Wolbers, 2014; Moffat et al., 2006; Wiener et al., 2013), especially in lower performing older adults (Schuck et al., 2015). Diersch et al. (2021) found that lower navigation performance and less spatial learning in older adults were accompanied by a disinhibition during navigation in the hippocampus, which did not change over learning trials.

Beyond its sensitivity to age-related changes, spatial navigation has also been suggested as a sensitive cognitive marker for pathological aging. For example, it is well established that Parkinson’s disease is associated with deficits of executive functions, working memory, and attention (Müller et al., 2000; Papagno & Trojano, 2018; Svenningsson et al., 2012), while recent evidence also points to spatial navigation deficits (Schneider et al., 2017; Thurm et al., 2016). In Alzheimer’s disease (AD), several studies have shown deficits in allocentric and egocentric navigation, especially in the translation between these perspectives (for review, Serino et al., 2014). Moreover, egocentric perspective rotation has been shown to differentiate types of dementia (Tu et al., 2015) and young- and late-onset AD (Pai & Yang, 2013). Research into preclinical markers of AD showed that AD risk carriers of the $\varepsilon$4 allele exhibited path integration deficits and a bias towards navigation in proximity to environmental boundaries, which was associated with grid cell instability (Bierbrauer et al., 2020; Kunz et al., 2015). Lastly, risk carriers showed longer mean wayfinding distance but no deficits in object-location long-term memory performance (Coughlan et al., 2019; Gellersen et al., 2021), and spatial navigation has been identified as a better cognitive marker than episodic memory for both preclinical AD (Allison et al., 2016) and AD progression (Levine et al., 2020). This indicates that navigation might be a more sensitive cognitive marker for AD than episodic memory tests.

To maximize the value of a navigation paradigm for the assessment of older participants and neurological patients, aspects of ecological validity and clinical feasibility must be balanced. Real-life active navigation tasks—e.g., the human analog of the Morris water maze (Majerová et al., 2012)—have the advantage of including vestibular and proprioceptive information, as well as motor efference copy signals. However, such large and complex setups are difficult to establish in clinical settings. In contrast, virtual environments hold great potential for assessments in complex and still-standardized environments with high ecological validity. However, more immersive virtual reality (VR) setups require specialized equipment and are associated with cybersickness symptoms such as nausea and headaches during navigation due to the discrepancy between visual and vestibular inputs (Diersch & Wolbers, 2019; Krohn et al., 2020). Desktop VR setups only require a two-dimensional (2D) screen and have been shown to reliably estimate real-world navigation performance while avoiding cybersickness (Hejtmanek et al., 2020). Both in immersive and desktop VR, active movement requires the use of an interface device (e.g., mouse, keyboard, or touch screens) [virtual spatial navigation assessment (Ventura et al., 2013); Sea Hero Quest (Coutrot et al., 2018; Spiers et al., 2016)], which adds complexity and training time, especially in older adults. Furthermore, both physical and virtual active navigation direct cognitive resources away from the task to prioritize control of walking (Simieli et al., 2015). To overcome these limitations, paradigms have been developed that use videos to passively transport participants along a given route with minimal [e.g., route-learning test suite by Wiener et al. (2020)] or no input from the participant during navigation [e.g., navigation test by van der Ham et al. (2020)].

Despite the wealth of knowledge acquired on different processes of spatial navigation, their neural basis, and the relevance of navigation for aging and age-related neuropathologies, no gold standard for the evaluation of spatial navigation has been established yet, especially with a focus on online spatial navigation. Although its complexity precludes a unified approach to a domain-specific test of spatial navigation, a specific and empirically validated test construct can facilitate translation to clinical settings and identify both specific impairments and preserved or less impaired types of navigation, even in patients with known deficits in episodic memory. The VIENNA spatial navigation assessment introduced here is intended to be applicable in experimental and clinical settings, and focuses on visuospatial and executive elements of navigation by providing online available spatial cues from egocentric and allocentric perspectives in increasingly complex unfamiliar environments. VIENNA is a desktop VR adaptation of a real-life video paradigm (Rekers & Niedeggen, 2022) and uses improved stimulus design to enable the detection of spatial updating and perspective rotation errors. Here, we first controlled for the effects of VR implementation in a pilot study by comparing a VR replication of the original real-life video paradigm and an improved VR adaptation. Next, we tested the feasibility of VIENNA in healthy middle-aged and older adults, and assessed its variability, item difficulty, and internal consistency. We then examined the validity of VIENNA outcome measures using self-report and neuropsychological tests. Hypotheses regarding convergent cognitive markers were a positive association with visuo-perceptive and constructive abilities, visuospatial short-term and working memory, mental rotation and perspective taking, as well as executive functions. In addition, we expected discriminant markers in the form of no or small associations with visual episodic memory, selective attention, and strategy application, due to the homogenized approach to the task. Lastly, we aimed to scrutinize the visuospatial and executive focus of VIENNA, considering relevant demographic variables, general cognitive performance, and the intercorrelation between relevant markers.

## Methods

We developed and evaluated a virtual adaptation of the previously designed navigation paradigm by Rekers and Niedeggen (2022), which relied on filmed hallways. Advantages of the virtual adaptation included a higher degree of control over stimulus design and an easier development of parallel versions. To this end, we conducted two studies: a pilot study, exploring the advantages of two different virtual versions of the original real-world paradigm in 31 volunteers, and the main study, where we thoroughly investigated the final VIENNA paradigm’s properties in a new sample of 79 middle-aged to older adults and compared VIENNA performance to self-report measures and other neuropsychological tests.

### Pilot study

To test the virtual implementation of the real-world paradigm, we first reconstructed the real-world hallways in a virtual environment accurately representing the layout of the original hallways (reconstructed version). Second, we adapted the original layout in a refined adaptation of this virtual reconstruction, where the hallway layouts were arranged more symmetrically to increase the requirements for accurate perspective rotation and spatial updating. These two versions—the reconstruction and the refined adaptation—were then compared in a pilot study in healthy older adults. In the pilot study, we tested 31 healthy seniors (21 female) with a mean age of 70.0 years ($SD$ = 6.1, range 62–85 years) in parallel-group testing conducted concurrently in two rooms. Random assignment resulted in 14 participants being assessed with the reconstructed version and 17 participants being tested with the refined adaptation of the paradigm.

### Participants

For the study using the final VIENNA paradigm, a priori power analysis based on the correlation between the previous version of the navigation paradigm and mental rotation performance with an effect size of $r$ = .30 indicated a required sample size of *n* = 82 ($\alpha$ = .05, power (1 − $\beta$) = .80). Allowing for an 8–10% attrition rate, 88 adults were recruited via local senior citizens’ clubs and internet advertisements. Participants received a report on their performance in the cognitive tests or financial compensation. A priori defined exclusion criteria included a history of neurological or acute psychiatric disorders; visuospatial perceptive deficits identified by a perspective translation pretest (Rekers & Niedeggen, 2022); general cognitive impairment as identified by a Mini-Mental State Examination [MMSE (Folstein et al., 1975)] score of < 24; and significant deficits (percentile < 10) in more than one of the following cognitive domains assessed in the neuropsychological assessment described below: visuoconstruction, episodic memory, selective attention, and executive functions. One participant was excluded due to a neurological disorder and eight participants were excluded due to significant deficits in more than one cognitive domain, resulting in a sample of *n* = 79 participants for statistical analyses (*n* = 21 male) with a mean age of 67.8 years ($SD$ = 8.8, range 50–85 years). All participants had normal or corrected-to-normal vision. Additional details on participants’ demographic information and self-report measures are provided in the Supplementary information in Table B1 for categorical and in Table B2 for at least ordinal variables. Written informed consent was obtained from all participants and the study was approved by the local ethics committee at Charité - Universitätsmedizin Berlin.

### Procedure

Participants were tested individually by trained psychologists and neuroscience students. First, participants filled out the Complainer Profile Identification [CPI (Lubitz et al., 2018)]; the German translation of the Santa Barbara Sense of Direction Scale [SBSOD (Hegarty et al., 2002)] called the Freiburg Santa Barbara Sense of Direction Scale [FSBSOD (Meilinger & Knauff, 2004)]; and the Geriatric Depression Scale [GDS (Yesavage & Sheikh, 1986)]. Beyond VIENNA, the neuropsychological examination included assessments of (1) visuoconstruction (copy trial, Rey-Osterrieth Complex Figure Test [ROCF (Rey, 1941)]); (2) visual episodic memory (delayed Rey-Osterrieth Complex Figure Test, percent recalled); (3) visuospatial short-term and working memory (block tapping, visual memory span forward and backward, Wechsler Memory Scale-Revised Version (Wechsler, 1987)); (4) screening for general cognitive abilities (Mini-Mental State Examination); (5) attention (selective subtest “auditiv I”, Testbatterie zur Aufmerksamkeitsprüfung [TAP (Zimmermann & Fimm, 2009)]) with median reaction time as a measure for psychomotor processing speed and omitted reactions as a measure for selective attention; (6) mental rotation (Vandenberg’s Mental Rotation Test [MRT (Vandenberg & Kuse, 1978)]); (7) visuospatial executive functions (Five-point Test: productivity, flexibility, and strategy [FPT (Goebel et al., 2009; Regard et al., 1982)]); and (8) (egocentric) perspective taking (Perspective Taking Test [PTSOT (Hegarty & Waller, 2004)]).

### Paradigm

**Fig. 1:**
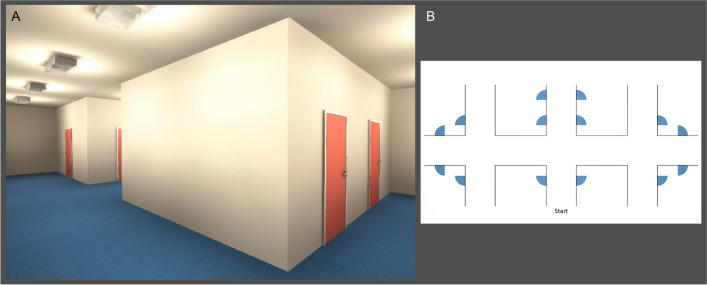
Illustration of the stimuli used in VIENNA. **A** Screenshot of the video shown during a trial of VIENNA. Participants watch the video of the hallway exploration, which is presented on the left side of the screen. **B** Allocentric map of the respective trial which is presented throughout the trial on the right side of the screen and used by the participant to indicate at which door the video ends

For the final VIENNA paradigm, we used the refined adaptation tested in the pilot study with two additional, more complex trials to avoid ceiling effects. VIENNA is a passive navigation assessment that relies on online available rather than retrieved (offline) spatial representations. To control for age-related deficits in the perception of speed and direction of self-motion cues, and considering that older adults tend to take more time to familiarize themselves with an environment (e.g., Diersch et al., 2021), we recorded videos at a reduced walking speed of the first-person character of 0.8 m/s instead of the default 1 m/s. Furthermore, instructions and stimuli are designed to homogenize the strategic approach to the task and thus the cognitive functions tested with it. Virtual environments and maps were created using Blender [Version 2.79b (Blender Online Community, 2018)] and Unity [Version 2018.1; Unity Technologies, Inc. San Francisco, CA, USA], and postprocessing of the videos was done in Adobe Premiere Pro CC 2018. The paradigm was created and run using the Python application PsychoPy3 (Peirce, 2007; Peirce et al., 2019). A schematic representation of the stimuli is presented in Fig. 1. VIENNA is freely available for researchers and clinicians at https://osf.io/kp4c5/.

VIENNA consists of one instruction trial, two practice trials, and 12 main trials. All trials show a first-person perspective of a character exploring virtual hallway environments. In addition, an allocentric map of the respective environment is displayed throughout each trial. Participants are required to mentally trace the character’s position and indicate the door that the character chose at the end of the trial. Importantly, this task design does not rely on episodic memory and does not require active exploration or navigation by the participant, thus homogenizing the available information to solve the task across participants. Details on the conceptual design of the paradigm are described in Rekers and Niedeggen (2022). The task involves translating ego- and allocentric perspectives using cues like boundaries and landmarks, spatial updating by extrapolating from egocentric movement to an allocentric path, and perspective rotation, when the egocentric perspective rotates while the allocentric representation remains in its original position.

The complexity of the trials is increased as the environments become larger and/or the character performs progressively more turns (trials 1 to 3: no-turn items; trials 4 to 6: single-turn items; trials 7 to 9: double-turn items; trials 10 to 12: one 180°-turn, full-turn items). Participants’ answers were documented by the examiner on answer sheets. Correct trials (i.e., correct door was chosen) were awarded two points. Trials with updating errors (i.e., choosing a door parallel or adjacent to the correct door) and trials with perspective rotation errors (i.e., choosing a door opposite to the correct door) were awarded one point (see Figure A1 for exemplary scoring). The total VIENNA score is the main outcome measure and was calculated across all 12 main items. As auxiliary measures, the number of updating and perspective rotation errors was recorded.

### Data analysis

We used the software environment $R$ [Version 3.6.1 (R Core Team, 2016)] for data preprocessing, quality check, and statistical analyses, and prepared the manuscript using the $R$ package $papaja$ [Version 0.1.0.9997 (Aust & Barth, 2020)]. Analysis scripts, including all packages and version information, are available at https://osf.io/kp4c5/. The level of significance was set at $p$ < .05 for all tests. In the pilot study, performance distribution, floor and ceiling effects, and differences in mean performance using a $t$-test for independent samples were compared between the virtual reconstruction and its refined adaptation. Dropouts and the presence of univariate outliers were used to assess VIENNA’s feasibility. To assess the psychometric properties of VIENNA items, we investigated the variability, floor and ceiling effects, and the difficulty ratio of empirical to theoretical difficulty ($\frac{\overline{M}}{E}$) and the corrected correlation of the items with the total test score. The empirical difficulty of an item is quantified by the mean performance in the sample ($\overline{M}$), which was then divided by the theoretical difficulty, quantified by the expected value ($E$) of an item, to account for differing numbers of doors and one-point answers. The corrected item total correlation was measured using Wilson’s $e$ (Wilson, 1974), which has been considered a more appropriate measurement of correlation for associations between ordinal variables with tied ranks and metric variables than Pearson’s product moment correlation (Eid et al., 2010). To test for unidimensionality, VIENNA’s internal consistency was assessed by estimating the polychoric correlation matrix and calculating the reliability coefficient, polychoric ordinal $\alpha$, from this matrix.

The following missings occurred, which have not been imputed due to the systematic nature of the missings: In the Geriatric Depression Scale, the scores of three participants are missing, since not all items were answered. Vandenberg’s Mental Rotation Test was not attempted by four participants, and one participant refused to attempt the Perspective Taking Test because they found the respective task too hard or did not understand what they had to do following the instructions. Furthermore, we retained the data of the 13 participants who completed less than half of all Perspective Taking Test trials. In the Five-point Test score flexibility, one outlier was identified using outlier detection for skewed distribution with adjusted boxplot (Hubert & Vandervieren, 2008) and this value was excluded from the analyses. To check for potential nonlinear relationships between variables, scatterplots of the correlation of demographic, questionnaire, and cognitive variables with the VIENNA test score were inspected. We assumed normal distribution of ordinal or metric variables when absolute $z$-scores of skewness and excess kurtosis were below a threshold for medium sample sizes of 3.29 (Kim, 2013).

Subsequently, correlation analyses of the test score with collected variables were conducted using Pearson’s product moment correlation ($r$) for normally distributed variables and Spearman’s rho (${r}_{s}$) for non-normally distributed variables. We used partial correlations to control for the effect of metric variables and compared effect sizes using a $Z$ score based on the comparison of standardized regression coefficients (Clogg et al., 1995). Given homogeneity of variance (Levene’s test), we assessed nominal variables with two categories using a $t$-test for independent samples. Post hoc analyses were then conducted to investigate the nature of relationships that reached statistical significance. Finally, we examined how much variance in the VIENNA test score could be explained by the collected variables, while accounting for intercorrelation between the predictors using a forced-entry multiple regression analysis. Here, we included domain-specific predictors that reached statistical significance on an individual level. At least ordinal predictors were approximated as metric and all predictors were entered in the model at the same time. To identify the most parsimonious model, we successively reduced predictors by excluding predictors with a $p$ value below .1 or $p$ < .05 if the liberal threshold of .1 did not result in a predictor reduction. We stopped eliminating predictors when the more parsimonious model explained significantly less variance than the model before reduction or when all predictors reached significance. To validate this approach, we compared the resulting model to a stepwise regression based on the Akaike information criterion (AIC) with backward selection.

## Results

### Pilot study

In the pilot study, we compared performance levels between the virtual reconstruction of the original paradigm to the refined adaptation of this virtual reconstruction, which uses symmetrical layouts. Performance levels in the virtual reconstruction ($\overline{M}$ = 16.86 ($SD$ = 3.08), $\widetilde{M}$ = 17.50 (MAD = 2.22), range 11–20) and the adaptation variant ($\overline{M}$ = 16.59 ($SD$ = 2.62), $\widetilde{M}$ = 17 (MAD = 2.97), range 11–20) were very similar and did not differ significantly ($t\left(29\right)=0.26$, $p=.795$). Looking at the distribution of the two versions, the adaptation was not inferior regarding skewness (−0.47) and excess kurtosis (−0.47) to the virtual reconstruction (skewness = −0.77, kurtosis = −0.93), and neither version produced floor effects in the sample. However, we found mild ceiling effects, with three participants scoring the highest possible score in the virtual reconstruction and two participants scoring the maximum 20 points in the adaptation. Considering its favorable attributes regarding symmetry and scoring, we decided to use the adaptation for the final paradigm and add two more items to counter ceiling effects. Since the number of items in the adaptation variant was not balanced per item type (no-turn: 3, single-turn: 2, double-turn: 2, and full-turn: 3), one more complex single-turn item and one more complex double-turn item were added to the adaptation for the final version of VIENNA.

### Feasibility and psychometric properties of VIENNA

Descriptive statistics of the spatial navigation paradigm are summarized in Table 1. VIENNA scores in the sample were normally distributed in the upper half of the theoretically possible test score range of 0 to 24. The distribution of the VIENNA scores and error types is portrayed in the Supplementary information in Figure B1. The distribution of perspective rotation errors was slightly right-skewed while updating errors showed significant right skewness, with only 10 participants making more than one updating error. The median VIENNA administration time, including pretest and instructions, was 16 min. The applied standardized instructions proved adequate to ensure that participants completed the task in accordance with the test concept and that no participant terminated the assessment prematurely. Furthermore, no outlier scoring below or above 2.5 standard deviations from mean VIENNA performance was observed.

**Table 1:** Descriptive statistics of the VIENNA scores and error types

	$\overline{M}$	$SD$	$\widetilde{M}$	MAD	Min	Max	$SE$	${\gamma }_{1}$	${z}_{{\gamma }_{1}}$	${\gamma }_{2}$	${z}_{{\gamma }_{2}}$
VIENNA score	19.43	2.61	19	2.97	13	24	0.29	−0.29	−1.05	−0.42	−0.78
Rotation errors	1.49	1.12	1	1.48	0	4	0.13	0.26	0.96	−0.80	−1.50
Updating errors	0.59	0.84	0	0.00	0	3	0.09	1.37	5.06	1.16	2.17

${\gamma }_{1}$ = skewness, ${z}_{{\gamma }_{1}}$ = $Z$ score skewness, ${\gamma }_{2}$ = excess kurtosis, ${z}_{{\gamma }_{2}}$ = $Z$ score excess kurtosis

Table 2 shows the psychometric properties of VIENNA items for each item type. To account for the differing numbers of doors and numbers of one-point doors in each item, we investigated the difficulty using the ratio of empirical to theoretical difficulty ($\frac{\overline{M}}{E}$), with higher values indicating easier trials. Although the ratio of empirical to theoretical difficulty was heterogeneous within the item types, and the first four items showed a small variance with ceiling effects in item 4, which was solved correctly by all participants, we observed a successive increase in difficulty across item types, i.e., an increase of average difficulty from 6.25 (no-turn items), 6.01 (single-turn items), 4.48 (double-turn items), to 2.38 (full-turn items).

**Table 2:** Properties of the VIENNA items

Item	Item type	$\frac{\overline{M}}{E}$	$\overline{M}$	$E$	0	1	2	${s}^{2}$	$e$
i1	No-turn	3.94	1.97	0.50	0	2	77	0.02	0.19
i2	No-turn	5.42	1.95	0.36	0	4	75	0.05	0.57
i3	No-turn	9.38	1.97	0.21	1	0	78	0.05	0.66
i4	Single-turn	6.67	2.00	0.30	0	0	79	0.00	0.00
i5	Single-turn	5.11	1.84	0.36	4	5	70	0.24	0.13
i6	Single-turn	6.24	1.81	0.29	0	15	64	0.16	0.21
i7	Double-turn	7.57	1.59	0.21	8	16	55	0.45	0.53
i8	Double-turn	3.19	1.37	0.43	20	10	49	0.75	0.33
i9	Double-turn	2.68	0.51	0.19	47	24	8	0.46	0.46
i10	Full-turn	1.91	1.53	0.80	9	19	51	0.48	0.39
i11	Full-turn	2.27	1.41	0.62	1	45	33	0.27	0.44
i12	Full-turn	2.96	1.48	0.50	8	25	46	0.46	0.31

Item difficulty ($\frac{\overline{M}}{E}$), quantified by the ratio of the mean performance in the sample ($\overline{M}$) to the expected value ($E$) given the number of doors and number of partially correct answers in the respective trial; the absolute frequency of 0, 1, and 2 points scored in the sample; the variance (${s}^{2}$) and corrected item-total correlation quantified by Wilson’s $e$

The corrected item-total correlation indicates a good correlation with the test score, operationalized by Wilson’s $e$ > .30, for 8 of the 12 items. A low discrimination of $e$ < .20 was found for items 1, 4, and 5. The polychoric ordinal alpha without item 4, which had no variance, indicated poor internal consistency (0.54, 95% CI [0.38, 0.68]). Next, we also investigated the internal consistency when only considering items with a variance of > 0.05 and found that the polychoric ordinal alpha indicated an internal consistency of 0.67 (items 5 to 12; 95% CI [0.55, 0.77]). Taken together, item difficulty and internal consistency indicate a potentially underlying factor structure. However, testing for multidimensionality was beyond the scope of this work and would have required a larger sample size and more item variance.

### Correlates of VIENNA performance

To identify correlates of VIENNA performance, we assessed its relationship to demographic information, questionnaire data, and neuropsychological tests. Table 3 summarizes the correlations and partial correlations accounting for participant age, and averaged overall cognitive performance, correcting for multiple comparisons. Descriptive statistics for questionnaire and neuropsychological data in the sample are summarized in the Supplementary information in Table B2 and Table B3. Scatterplots of all significant correlations with the VIENNA score are portrayed in the Supplementary information in Figure C1 and Figure C2.

**Table 3:** Correlations of VIENNA with demographic, questionnaire, and cognitive variables

Variable	$r$	${r}_{XV\cdot age}$	${r}_{XV\cdot \overline{X}}$
Age	−.54***	-	-
Years of education	.18	-	-
CPI	−.13	-	-
CPI: memory	−.14	-	-
CPI: attention	−.15	-	-
CPI: executive	−.05	-	-
FSBSOD	.37**	-	-
GDS _s_	−.04	-	-
MMSE _s_	.46***	.37**	.38**
ROCF copy _s_	.36**	.34**	.17
ROCF delayed recall %	.24	.12	−.01
Block span forward	.38**	.33**	.04
Block span backward	.51***	.39**	.18
TAP: Processing speed	.11	.18	.17
Vandenberg MRT	.41***	.26*	−.03
PTSOT	.57***	.52***	.35*
FPT productivity	.52***	.42***	.33*
FPT flexibility _s_	.10	.06	−.04
FPT strategy	.26*	.16	.06
Average performance	.67***	-	-

$r$ = Pearson correlation coefficients, ${r}_{XV\cdot age}$ = partial correlations correcting for age, ${r}_{XV\cdot \overline{X}}$ = partial correlations correcting for average performance in neuropsychological tests. _s_ = Spearman’s rho, *** < .001, ** < .01, * < .05, significance levels after correcting for multiple comparisons using Benjamini–Hochberg. CPI = Complainer Profile Identification, FSBSOD = Freiburg Santa Barbara Sense of Direction Scale, GDS = Geriatric Depression Scale, MMSE = Mini-Mental State Examination, ROCF = Rey-Osterrieth Complex Figure Test, TAP = Testbatterie zur Aufmerksamkeitsprüfung, MRT = Mental Rotation Test, PTSOT = Perspective Taking Test, FPT = Five-point Test

#### Demographic and questionnaire data

Of all demographic variables assessed, only age proved to be significantly associated with VIENNA performance. We found a large negative correlation with age, with a steeper decline in performance after the age of 60. We did not observe effects of gender (male vs. female participants; $t\left(77\right)=-0.38$, $p=.702$) or reported years of education ($p$ = .189), and men and women did not differ in age ($t\left(77\right)=1.26$, $p=.213$). With respect to self-report measures, we did not find a significant correlation with the overall measure of cognitive complaints ($p$ = .324) or with the Complainer Profile Identification subscores memory ($p$ = .314), attention ($p$ = .262), and executive function ($p$ = .717). Subjective sense of direction, measured using the Freiburg Santa Barbara Sense of Direction Scale, showed a medium-sized positive correlation with the VIENNA score, whereas depressiveness, assessed by the Geriatric Depression Scale, did not correlate significantly with the VIENNA score ($p$ = .717). Taken together, age proved to be the only demographic variable, and subjective sense of direction the only self-report measure, with a significant relationship with the VIENNA performance.

#### Neuropsychological components of VIENNA

To investigate the construct validity of VIENNA, we assessed its relationship with standard neuropsychological assessments for visuospatial abilities, visual episodic memory, short-term and working memory, visual executive functions, and attention, as well as with the Mini-Mental State Examination. Given the significant effect of age, we also assessed partial correlations correcting for age. Lastly, we calculated the partial correlations correcting for the average performance in all other neuropsychological tests except for VIENNA and the respective test. All effect sizes with $p$ values corrected for multiple comparisons are reported in Table 3.

VIENNA performance correlated significantly with the following tests: (i) large correlations with Mini-Mental State Examination, visuospatial working memory, perspective taking, and visuoconstructive productivity; (ii) medium to large correlation with mental rotation; (iii) medium correlations with visuoconstruction and visuospatial short-term memory; (iv) and small to medium correlation with the executive function strategy application. VIENNA did not correlate significantly with episodic memory, operationalized by the percentage of recalled elements in the delayed free recall of the Rey-Osterrieth Complex Figure Test ($p$ = .059), cognitive flexibility ($p$ = .407) or attention regarding processing speed ($p$ = .407), and selective attention ($t\left(75\right)=0.18$, $p=.857$).

Correcting for participant age, all previously significant correlations, except for strategy application, remained significant, and effect sizes for correlations with working memory ($Z$ = 1.11), productivity ($Z$ = 1.05), and mental rotation ($Z$ = 1.20) decreased significantly. When correcting for average performance in neuropsychological tests, only the correlations with Mini-Mental State Examination, perspective taking, and visuoconstructive productivity remained significant and showed medium-sized effects.

In order to test the validity of the error types *perspective rotation* and *updating* in VIENNA, we assessed the correlation between perspective rotation errors and the Perspective Taking Test and Vandenberg’s Mental Rotation Test and the relationship between updating errors and the block span tasks for short-term and working memory. Perspective rotation errors correlated significantly with the Perspective Taking Test ($r=-.35$, 95% CI $\left[-.53,-.13\right]$, $t\left(76\right)=-3.21$, $p=.002$), but not with the mental rotation task ($t\left(73\right)=-1.14$, $p=.260$). The correlation with the Perspective Taking Test had a medium effect size and indicated that a higher number of perspective rotation errors was associated with a worse performance in the Perspective Taking Test. Updating errors did not correlate significantly with short-term ($S=93,210.51$, $p=.237$) or working memory ($S=97,197.24$, $p=.106$). However, considering the small variance of updating errors, post hoc groups comparisons were performed that showed that participants with three updating errors compared to participants without or with one updating error performed significantly worse in visuospatial short-term memory ($p$ = .047) and working memory ($p$ < .001 and $p$ = .002). Visualizations can be found in the Supplementary information in Figure C3.

#### Relationship among the variables

Based on recent findings by van der Ham et al. (2021), we assessed the impact of age and gender on the relationship between the Freiburg Santa Barbara Sense of Direction Scale and VIENNA. Multiple regression analyses with interacting predictors showed that age did not correlate significantly with self-report ($t\left(75\right)=1.64$, $p=.106$) or interact with the significant relationship between Freiburg Santa Barbara Sense of Direction Scale and VIENNA performance ($t\left(75\right)=-1.55$, $p=.125$). However, men reported a significantly better sense of direction than women ($b=3.27$, 95% CI $\left[0.99,5.54\right]$, $t\left(75\right)=2.86$, $p=.005$), although men and women did not significantly differ in their navigation performance ($t\left(77\right)=-0.38$, $p=.702$). Indeed, being male had a negative impact on the correlation between self-reported and measured navigation ability ($b=-0.14$, 95% CI $\left[-0.25,-0.02\right]$, $t\left(75\right)=-2.38$, $p=.020$).

Multiple regression analyses of the predictors of VIENNA performance were conducted in a subset of 74 participants without missing data. Model 1 included all nine domain-specific predictors that reached significance on single level and explained 59% of the variance in the VIENNA performance (${R}^{2}=.59$, $F\left(9,64\right)=10.05$, $p<.001$). In model 2, we included the four predictors from model 1 with a $p$ value below .1. The model which included age, visuoconstruction, perspective taking, and visual productivity explained 56% of the variance in the VIENNA performance (${R}^{2}=.56$, $F\left(4,69\right)=21.82$, $p<.001$). Model 2 was considered the most appropriate model, since all predictors reached statistical significance, and comparing models 1 and 2 in an analysis of variance (ANOVA), model 2 did not explain significantly less variance ($\Delta {R}^{2}$ = 0.03, $F$(−5, 69) = 0.84, $p$ = .527). Furthermore, a stepwise regression based on the AIC with backward selection confirmed this model. The results of model 2 are portrayed in Table 4, and model 1 is portrayed in the Supplementary information in Table C1.

**Table 4:** Multiple regression model 2, explaining 56% of the variance in VIENNA performance

Predictor	$b$	95% CI	$t$(69)	$p$
Intercept	19.51	[19.10, 19.93]	94.14	< .001
Age	−0.94	[−1.40, −0.49]	−4.13	< .001
ROCF copy	0.53	[0.09, 0.98]	2.41	.019
PTSOT	0.74	[0.24, 1.24]	2.93	.005
FPT productivity	0.58	[0.10, 1.06]	2.41	.019

ROCF = Rey-Osterrieth Complex Figure Test, PTSOT = Perspective Taking Test, FPT = Five-point Test

**Fig. 2:**
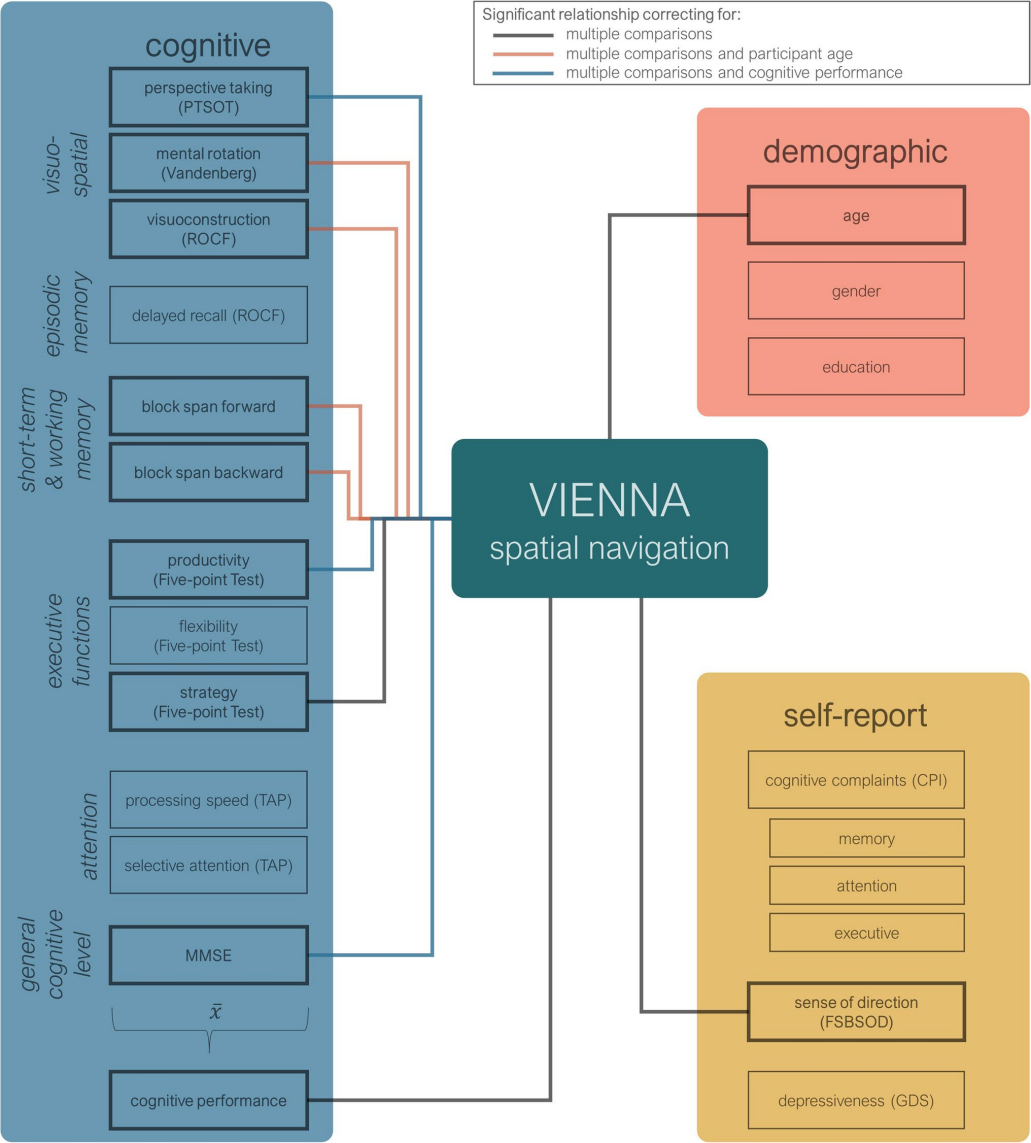
Cognitive, demographic, and self-report correlates of the VIENNA score. PTSOT = Perspective Taking Test, ROCF = Rey-Osterrieth Complex Figure Test, TAP = Testbatterie zur Aufmerksamkeitsprüfung, MMSE = Mini-Mental State Examination, CPI = Complainer Profile Identification, FSBSOD = Freiburg Santa Barbara Sense of Direction Scale, GDS = Geriatric Depression Scale

Figure 2 gives an overview of the predictors of VIENNA performance. Overall, we found evidence for convergent validity of VIENNA in its correlations with self-reported sense of direction and with perspective taking, visuoconstruction, mental rotation, visuospatial short-term and working memory, and productivity. The a priori hypotheses regarding executive functions were not confirmed for strategy application, which correlated with VIENNA performance but was not robust to age correction. VIENNA did not correlate with visual episodic memory or selective attention, supporting discriminant validity. Furthermore, VIENNA performance showed a very stable correlation with Mini-Mental State Examination scores and was sensitive to age but not to gender, education, depressiveness, or subjective cognitive complaints in the domains memory, attention, or executive functions.

## Discussion

We introduce VIENNA, a brief, passive desktop VR paradigm for evaluating spatial navigation beyond episodic memory with minimal technical and motor demands. We show that VIENNA performance reflects visuospatial and executive components of navigation, is sensitive to age, and is associated with self-reported sense of direction. Furthermore, we found high feasibility for middle-aged and older adults, with an average application time of 16 min and a successive increase in difficulty across the navigation test. These features of VIENNA allow for a translation of spatial navigation assessment from experimental to clinical settings.

VIENNA aims to objectify and operationalize the everyday challenge in navigating novel environments using an analog or digital map. Such navigation deficits significantly limit people’s autonomy. However, these deficits are often not immediately apparent to a person’s social network since they may manifest subtly, for instance, as reluctance or fear to move independently in new surroundings (Burns, 1999; Davis & Veltkamp, 2020). From a clinical perspective, the focus on visuospatial and executive components of navigation in new and dynamic environments can facilitate a differentiation between episodic memory deficits and spatial navigation deficits, since assessments relying predominantly on an episodic memory approach to navigation might generalize more specific deficits in one aspect relevant for navigational ability to spatial navigation in general. This construct specificity further serves to clarify which type of navigation performance and associated domains we are measuring. In addition, VIENNA was developed with complexity levels suitable for application in clinical populations and accommodates common motor impairments, such as hemiparesis, since participants do not need to navigate using an input device. Importantly, its short administration time of around 16 min is comparable to other commonly used neuropsychological tests like the Rey Auditory Verbal Learning Test (Schmidt, 1996). Therefore, VIENNA can be particularly well integrated into the limited examination times allotted for neuropsychological assessments in clinical settings.

VIENNA performance was associated with cognitive markers of visuoconstruction, visuospatial short-term and working memory, mental rotation, perspective taking, strategy application, and visuoconstructive productivity. These findings corroborate the importance of visuospatial processing, spatial updating, perspective rotation, and executive functions for the novel spatial navigation paradigm. This was also confirmed in analyses accounting for average performance across neuropsychological tests, where Perspective Taking Test, Five-point Test productivity, and Mini-Mental State Examination remained significant correlates, and in regression analyses accounting for the intercorrelation of all significant, domain-specific correlates, where age, visuoconstruction, Perspective Taking Test, and Five-point Test productivity were particularly relevant for VIENNA performance. The distinct contribution of productivity (i.e., the Five-point Test main outcome and measure of visuoconstructive fluency) can be explained by its role in spatial updating through sustained mental operation on changing stimuli, planning, abstract thinking, and rapid information processing. Perspective taking plays an essential role in VIENNA performance since increasing the number of rotations is the main mechanism to add difficulty between VIENNA items. The robust relationship of VIENNA performance with the Mini-Mental State Examination should be interpreted with caution, because of its small variance and since it assesses different cognitive domains using few items that are poor predictors of domain-specific performance (Moafmashhadi & Koski, 2013; Roebuck-Spencer et al., 2017). It could, however, indicate VIENNA’s sensitivity to subclinical cognitive impairments. We aimed to homogenize the strategic approach to VIENNA but, contrary to our expectations, found a small to medium correlation between VIENNA performance and strategy application. However, this does not necessarily indicate that different strategies lead to better VIENNA performance and could be explained by the robust relationship of both VIENNA and Five-point Test strategy with Five-point Test productivity (Goebel et al., 2009).

We found a large negative correlation between age and VIENNA performance. Controlling for participant age significantly decreased VIENNA’s correlations with executive markers visuospatial working memory, productivity, and strategy application, which is in line with results using the previous version of the paradigm (Rekers & Niedeggen, 2022) and with findings that executive processes in navigation are particularly sensitive to age-related decline (Taillade et al., 2013). Although mental rotation and perspective taking both decline with age, especially in speed tests (Dror & Kosslyn, 1994; Inagaki et al., 2002; Zancada-Menendez et al., 2016), only VIENNA’s association with mental rotation decreased when controlling for age, supporting the unique cognitive resources required by perspective taking for spatial navigation beyond mental rotation (Kozhevnikov et al., 2006).

We did not find associations of VIENNA performance with cognitive flexibility, selective attention, or visual episodic memory. Five-point Test flexibility, operationalized by the percentage of perseverations, may not have shown a relationship with navigation performance because VIENNA does not capture perseverative behavior during active navigation, which has been observed in older adults (Moffat et al., 2007). Furthermore, the absence of associations with TAP psychomotor reaction time or selective attention markers suggests that slow movement in the videos and untimed responses were successful in decoupling VIENNA performance from basic attention and speed parameters. Importantly, we did not find evidence for a correlation of VIENNA performance with visual episodic memory. This indicates that using online available spatial information in dynamic and unfamiliar environments successfully reduced the load on episodic memory in this task. This is supported by evidence from patients with profound hippocampal damage that show severe episodic memory deficits but intact navigation performance when maps are provided (Urgolites et al., 2016).

Age was the only demographic variable relevant for VIENNA performance. This is in line with the robust evidence of the negative effects of aging on spatial navigation (Lester et al., 2017; Moffat, 2009), especially regarding dynamic elements of navigation (van der Ham & Claessen, 2020). Although most navigation studies have shown a male advantage with a small-to-medium effect size (Nazareth et al., 2019), we did not find an effect of gender. While there were no systematic age differences between men and women in this study, the effect could be masked by a decrease of gender difference in spatial abilities with age (Jansen & Heil, 2009) and stronger aging effects of route navigation in men than in women (van der Ham et al., 2020). Education did not affect VIENNA performance in our participants. Previous research in a large international sample found a small effect of education on wayfinding performance (Coutrot et al., 2022), which might be greater in clinical populations considering the mitigating effect of education as a measure of cognitive reserve on functional impairments (Stern, 2009).

We showed that VIENNA performance is significantly associated with self-reported sense of direction, indicating that it objectifies subjective navigation ability. Scrutinizing the relationship of self-reported and measured spatial navigation ability, we observed an overestimation of ability by men, consistent with previous findings by van der Ham et al. (2021). In contrast to their results, the quality of self-report did not decrease with age, presumably because we did not include younger adults. Furthermore, VIENNA showed no association with cognitive complaints in memory, attention, or executive functions, which is in line with spatial navigation being distinct from these cognitive domains (Fan et al., 2021; Laczó et al., 2017). Particularly advantageous for clinical settings, VIENNA performance was not sensitive to subclinical depressive symptoms which often confound cognitive performance, especially in the domains of attention, executive functions, and memory (Culpepper et al., 2017). It is also consistent with other visuospatial tests, like the Clock Drawing Test, which has been shown to differentiate cognitive impairment due to depression and neurological disorders like Alzheimer’s disease (Herrmann et al., 1998).

In the pilot study, we found that the implementation of the hallways in virtual environments resulted in higher test scores, possibly due to distractor control and homogenized visual features, removing competition between irrelevant and relevant information (Montello, 2005; Weisman, 1981). Adding two more complex trials in the final version resulted in a normal distribution of VIENNA performance in the upper range of scores without ceiling or floor effects, allowing acquisition of potentially lower performance in patients with cognitive impairment. Furthermore, we did not observe dropouts or outliers, indicating that test and instruction design were intuitive and did not negatively impact performance. Moreover, the successive increase in item difficulty across item types avoids boredom and frustration when examining participants with diverse cognitive abilities. We also found a good correlation with VIENNA’s total score for eight out of 12 items, but only poor to acceptable internal consistency, probably due to the different item complexities. Alternatively, this could be explained by an underlying factor structure, especially with regard to no-turn items, which involve minimal perspective rotation and mainly use vista space as opposed to environmental space in other items. Although we did not formally assess inter-rater reliability, scoring of VIENNA performance is highly objective given the unambiguous evaluation procedure.

The validity and specificity of VIENNA’s perspective rotation errors was confirmed since they were significantly associated with perspective taking but not mental rotation, which is in line with their conceptualization and findings that the egocentric transformation of viewpoints is distinct from imagined spatial transformation of objects (Hegarty & Waller, 2004; Zacks et al., 2003). Updating errors occurred relatively seldom in our healthy participants, and we did not find a correlation with short-term or working memory. However, because individuals with the most (three) updating errors performed significantly worse in a spatial working memory task than participants with no or one updating error, the study could be underpowered given the distribution of this error type, with a power calculation assuming a power of 0.8 suggesting a required sample size of *n* = 189. While VIENNA’s error types should not replace domain-specific tests, perspective rotation and spatial updating errors can be useful auxiliary outcome measures to identify cases where more discrete impairments affect navigation performance.

This study has some shortcomings with respect to the neuropsychological assessments. Vandenberg’s Mental Rotation Test and the Perspective Taking Test were perceived as quite difficult by most participants and some refused to attempt the tasks, resulting in few but systematic missings. While this limits the generalizability of the results, it also emphasizes the need for more intuitive visuospatial rotation and especially navigation tasks. Furthermore, the association with episodic memory as one of the main outcomes was not subjected to multimodal testing, since the visuospatial focus of the test protocol limited testing time where no visual stimuli would interfere with consolidation. We also did not include a verbal episodic memory assessment because it is likely less closely related to VIENNA performance than visual memory, considering the conceptual distance.

Furthermore, to maximize clinical applicability, VIENNA only presents visual cues and does not include vestibular or proprioceptive feedback, as do more elaborate and resource-intensive paradigms involving physical movement by the participant (e.g., Iggena et al., 2022; Němá et al., 2021; Stangl et al., 2020). This affects the proximity to real-world navigation and may amplify age-related differences in navigation (Allen et al., 2004; Bates & Wolbers, 2014; Jabbari et al., 2021; Stangl et al., 2020; Xie et al., 2017). Moreover, the visuospatial and executive focus entails that users need to ensure that navigation deficits measured with VIENNA are not primarily due to underlying deficits in spatial perception or executive function impairments. To screen for basic impairment in the ability to extrapolate 3D spatial representations from a 2D screen, we provide a screening tool that can be applied before VIENNA (Perspective Translation Test, https://osf.io/4h65p/). To account for age-related differences in VIENNA performance, we are building appropriate normative data. Lastly, VIENNA was developed specifically for middle-aged and older adults, and ceiling effects would occur in younger adults. We therefore developed an adaptation of VIENNA (VIENNA Young, https://osf.io/4h65p/) with more complex items, which is currently being assessed.

Future studies will provide normative data and further psychometric information on VIENNA. While the normal distribution of VIENNA performance makes an application in research settings particularly interesting, it also requires larger normative samples to evaluate clinically relevant scores. We will also assess a potentially underlying factor structure, which could not be assessed in this study due to the small variance in some items. Furthermore, we will evaluate VIENNA’s test-retest reliability using parallel versions to avoid practice effects and ensure its applicability as an outcome measure in intervention studies. To this end, VIENNA’s feasibility and sensitivity as a marker of functional impairment in different neuropathologies will need to be assessed, which could also indicate associated brain networks. Regions in the medial temporal lobe, specifically the hippocampus, will likely be relevant in processing allocentric perspective and environmental novelty, associated with the mid-posterior hippocampus (Kaplan et al., 2014). In addition, VIENNA might be particularly sensitive to brain disorders affecting prefrontal, fronto-striatal, parietal, and retrosplenial networks due to their role in online and route navigation in new environments (e.g., Goodroe et al., 2018; Mitchell et al., 2018; Patai & Spiers, 2021), but we believe it can be broadly applied in cognitive neurology.

VIENNA is a concise spatial navigation paradigm which measures behavior relevant to everyday life and is intuitive to administer, objective, informative, and valid. Its versatile applicability using passive navigation in virtual environments makes it specifically feasible for a differential assessment of spatial navigation in older adults and clinical populations. VIENNA assesses navigation in unfamiliar environments using spatial information, available online, from both egocentric and allocentric perspectives, thus reducing the cognitive load on episodic memory. The gradually increasing item difficulty allows to capture performance in lower- and higher-performing individuals. VIENNA’s associations with self-reported measures indicate that it reflects subjective navigation ability, while being robust to depressiveness, cognitive complaints, and education. A thorough assessment of the VIENNA’s construct validity confirmed its focus on visuospatial and executive functions, and showed a particular sensitivity to age, visuoconstruction, perspective taking, and visuoconstructive productivity. Future studies will evaluate VIENNA’s clinical feasibility and discriminatory value for different neurological disorders, as well as the retest reliability using parallel versions. In summary, VIENNA provides a rapid and ecologically valid spatial navigation assessment in middle-aged and older participants and holds great potential to capture distinct navigation deficits in patients with neurological disorders.

### Supplementary Information

Below is the link to the electronic supplementary material.

Supplementary file1 (DOCX 670 KB)
