# Supplementary material for: Translating spatial navigation evaluation from experimental to clinical settings: The virtual environments navigation assessment (VIENNA)
Source: Behav Res Methods. 2023 May 11;56(3):2033–48. doi: 10.3758/s13428-023-02134-0 (PMC10991013; doi:10.3758/s13428-023-02134-0)
Supplement: Supplementary file 1 — Supplementary file1 (DOCX 670 KB) [file 13428_2023_2134_MOESM1_ESM.docx]

# Supplementary information

# Appendix A. Scoring and error types


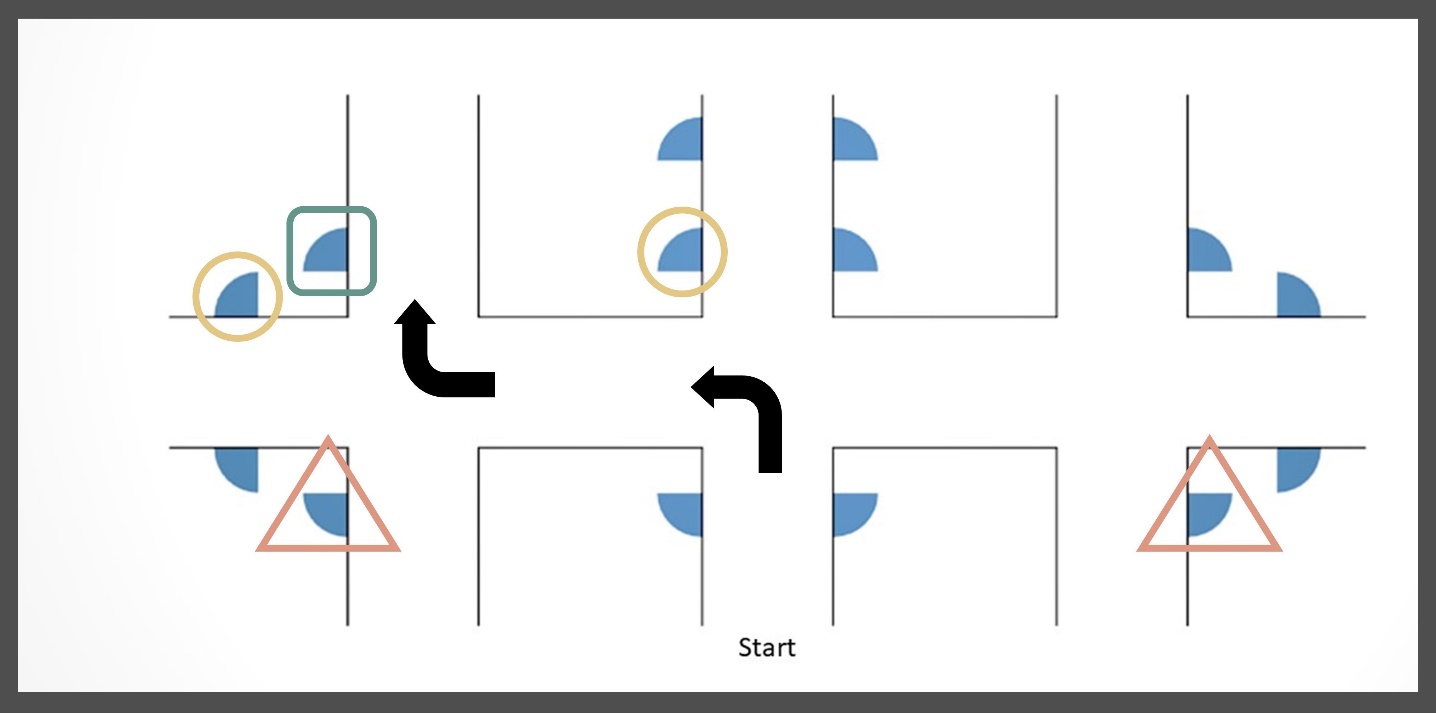
 **Figure A1.** Illustration of the scoring of VIENNA trials. Beyond the correct door (marked by green rectangle), mirrored layouts of the VIENNA trials allow to identify updating (yellow circle) and rotation errors (red triangle), i.e., one of the rotations in this trial, illustrated by the arrow, has not been identified correctly.

# Appendix B. Distribution of the applied measures in the sample


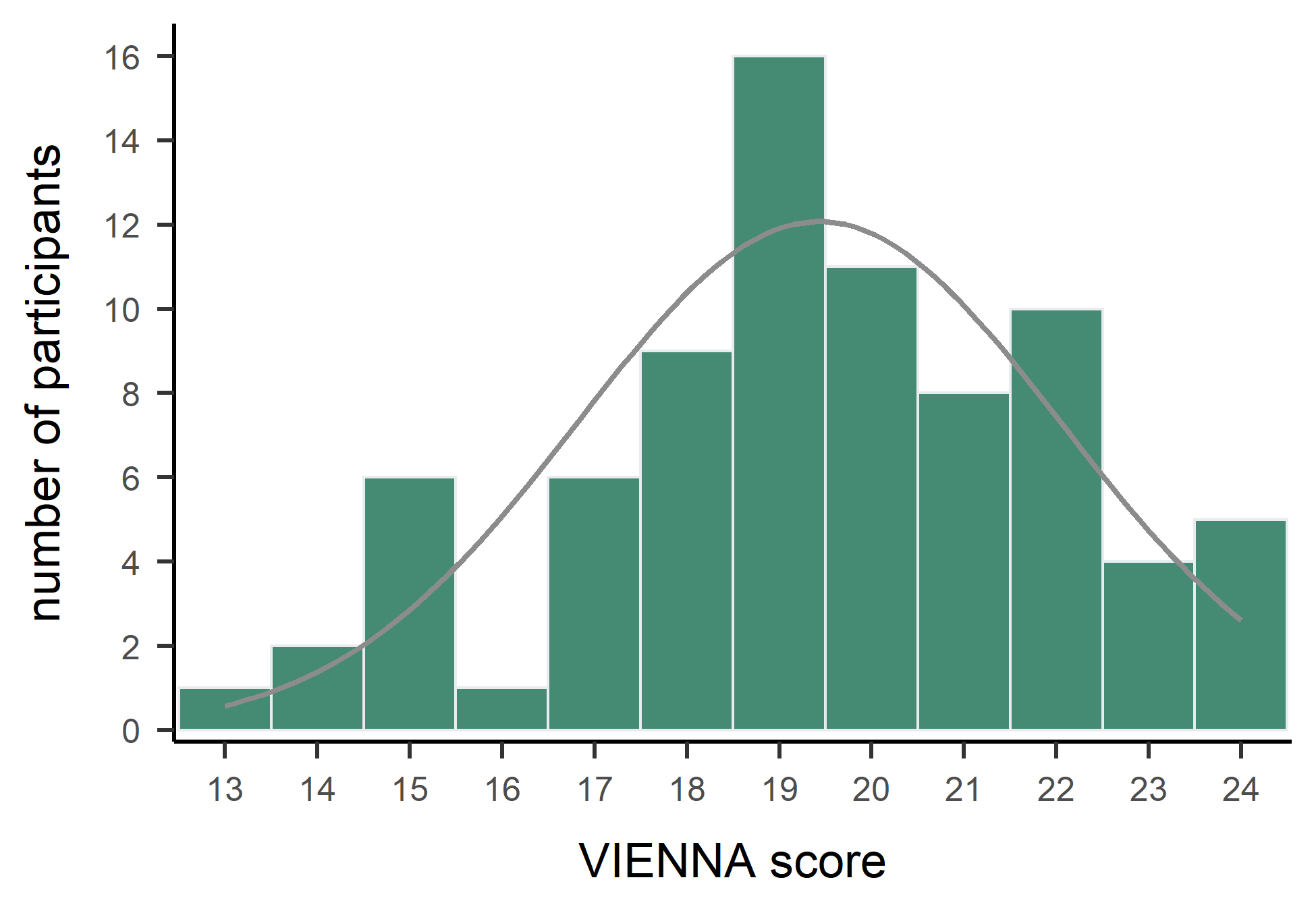

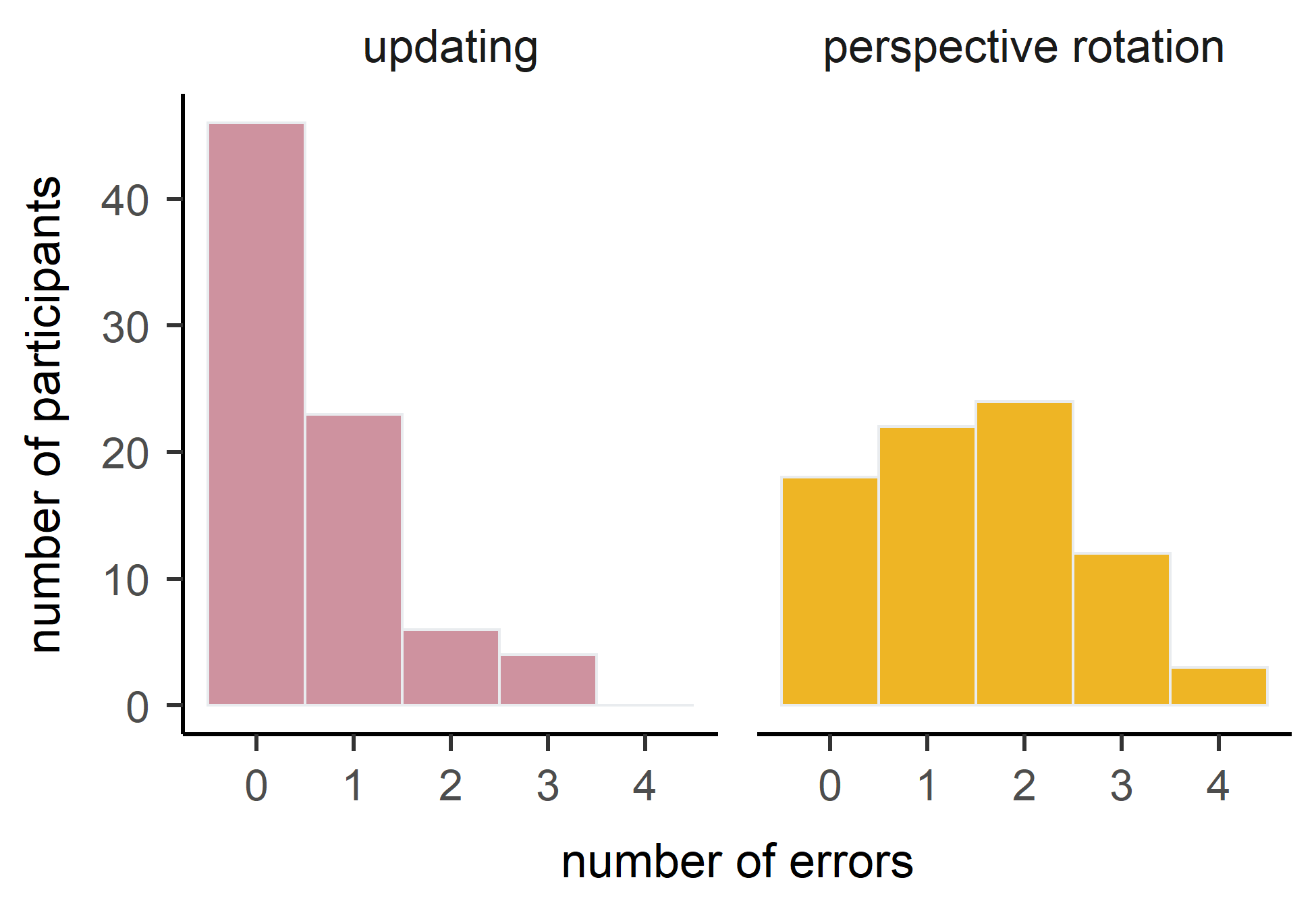


**Figure B1.** Histograms of the frequency distributions of VIENNA test scores (left) and the error types updating and perspective rotation.

**Table B1.** Summary of categorical demographic information in the sample.

| Variable | Level | N | % |
| --- | --- | --- | --- |
| Gender | female | 58 | 73 |
|  | male | 21 | 27 |
| Occupation | pensioner | 55 | 70 |
|  | full-time | 8 | 10 |
|  | unemployed | 6 | 8 |
|  | other | 4 | 5 |
|  | part-time | 4 | 5 |
|  | homemaker | 2 | 3 |
| Native language | German | 77 | 97 |
|  | other | 2 | 3 |
| Household | alone | 48 | 61 |
|  | not alone | 31 | 39 |
| Handedness | right | 71 | 90 |
|  | retrained | 6 | 8 |
|  | ambidextrous | 2 | 3 |

*Note.* Retrained participants were retrained from left- to right-handedness as children.

**Table B2.** Descriptives of at least ordinal demographic and questionnaire variables.

| Variable | $n$ | $\overline{M}$ | $SD$ | $\tilde{M}$ | MAD | Min | Max | Range | $SE$ | $\gamma_{1}$ | $z_{\gamma_{1}}$ | $\gamma_{2}$ | $z_{\gamma_{2}}$ |
| --- | --- | --- | --- | --- | --- | --- | --- | --- | --- | --- | --- | --- | --- |
| Age | 79 | 67.81 | 8.75 | 69.00 | 7.41 | 50.00 | 85.00 | 35.00 | 0.98 | -0.14 | -0.52 | -0.70 | -1.30 |
| Years of education | 79 | 15.93 | 2.52 | 16.50 | 2.22 | 10.00 | 19.00 | 9.00 | 0.28 | -0.67 | -2.47 | -0.67 | -1.26 |
| CPI | 79 | 1.35 | 0.54 | 1.29 | 0.61 | 0.38 | 2.71 | 2.33 | 0.06 | 0.37 | 1.36 | -0.45 | -0.83 |
| CPI: memory | 79 | 1.47 | 0.64 | 1.43 | 0.67 | 0.25 | 3.13 | 2.88 | 0.07 | 0.47 | 1.73 | 0.01 | 0.02 |
| CPI: attention | 79 | 1.33 | 0.59 | 1.29 | 0.62 | 0.29 | 3.00 | 2.71 | 0.07 | 0.41 | 1.53 | -0.16 | -0.30 |
| CPI: executive | 79 | 1.26 | 0.62 | 1.17 | 0.74 | 0.00 | 2.83 | 2.83 | 0.07 | 0.26 | 0.96 | -0.61 | -1.13 |
| FSBSOD | 79 | 3.63 | 0.76 | 3.53 | 0.79 | 1.60 | 5.40 | 3.80 | 0.09 | 0.07 | 0.27 | -0.48 | -0.90 |
| GDS | 76 | 1.71 | 2.15 | 1.00 | 1.48 | 0.00 | 11.00 | 11.00 | 0.25 | 1.95 | 7.19 | 4.75 | 8.88 |

*Note.* $\gamma_{1}$ = skewness, $z_{\gamma_{1}}$ = $Z$ score skewness, $\gamma_{2}$ = excess kurtosis, $z_{\gamma_{2}}$ = $Z$ score excess kurtosis. CPI = Complainer Profile Identification, FSBSOD = Freiburg Santa Barbara Sense of Direction Scale, GDS = Geriatric Depression Scale.

**Table B3.** Descriptives of cognitive variables besides VIENNA and overall standardized mean performance.

| Variable | $n$ | $\overline{M}$ | $SD$ | $\tilde{M}$ | MAD | Min | Max | Range | $SE$ | $\gamma_{1}$ | $z_{\gamma_{1}}$ | $\gamma_{2}$ | $z_{\gamma_{2}}$ |
| --- | --- | --- | --- | --- | --- | --- | --- | --- | --- | --- | --- | --- | --- |
| MMSE | 79 | 28.91 | 1.21 | 29.00 | 1.48 | 25.00 | 30.00 | 5.00 | 0.14 | -1.29 | -4.76 | 1.37 | 2.55 |
| ROCF copy | 79 | 35.05 | 1.83 | 36.00 | 0.00 | 26.00 | 36.00 | 10.00 | 0.21 | -2.86 | -10.57 | 9.04 | 16.90 |
| ROCF delayed recall % | 79 | 54.02 | 16.28 | 52.86 | 19.06 | 13.89 | 86.11 | 72.22 | 1.83 | -0.05 | -0.20 | -0.67 | -1.25 |
| Block span forward | 79 | 8.09 | 1.42 | 8.00 | 1.48 | 6.00 | 11.00 | 5.00 | 0.16 | 0.32 | 1.18 | -0.77 | -1.44 |
| Block span backward | 79 | 7.20 | 1.80 | 7.00 | 1.48 | 4.00 | 12.00 | 8.00 | 0.20 | 0.46 | 1.68 | -0.43 | -0.80 |
| TAP: Processing speed | 79 | 479.24 | 93.29 | 476.00 | 85.99 | 220.00 | 658.00 | 438.00 | 10.50 | -0.52 | -1.91 | -0.04 | -0.07 |
| Vandenberg MRT | 75 | 13.13 | 10.63 | 11.00 | 8.90 | -6.00 | 44.00 | 50.00 | 1.23 | 0.78 | 2.87 | 0.17 | 0.32 |
| PTSOT | 78 | 129.35 | 30.03 | 136.78 | 27.76 | 38.33 | 169.92 | 131.59 | 3.40 | -0.83 | -3.08 | -0.10 | -0.19 |
| FPT productivity | 79 | 27.96 | 6.01 | 28.00 | 4.45 | 7.00 | 43.00 | 36.00 | 0.68 | -0.17 | -0.63 | 1.30 | 2.44 |
| FPT flexibility* | 78 | 53.97 | 6.25 | 55.65 | 6.45 | 31.43 | 60.00 | 28.57 | 0.71 | -1.26 | -4.67 | 1.36 | 2.54 |
| FPT strategy | 79 | 40.64 | 18.87 | 44.44 | 24.35 | 4.55 | 70.97 | 66.42 | 2.12 | -0.20 | -0.73 | -1.29 | -2.41 |
| Overall performance | 79 | 0.00 | 0.52 | -0.05 | 0.51 | -1.14 | 1.19 | 2.33 | 0.06 | 0.13 | 0.48 | -0.33 | -0.62 |

*Note.* ${}^{*}$ Outlier detected in visual inspection, set to NA. $\gamma_{1}$ = skewness, $z_{\gamma_{1}}$ = $Z$ score skewness, $\gamma_{2}$ = excess kurtosis, $z_{\gamma_{2}}$ = $Z$ score excess kurtosis. MMSE = Mini-Mental State Examination, ROCF = Rey-Osterrieth Complex Figure Test, TAP = Testbatterie zur Aufmerksamkeitsprüfung, MRT = Mental Rotation Test, PTSOT = Perspective Taking Test, FPT = Five-point Test. TAP, PTSOT, and FPT flexibility are inverted so that high value indicates good performance.

# Appendix C. Additional information about VIENNA outcomes in relation to other measures


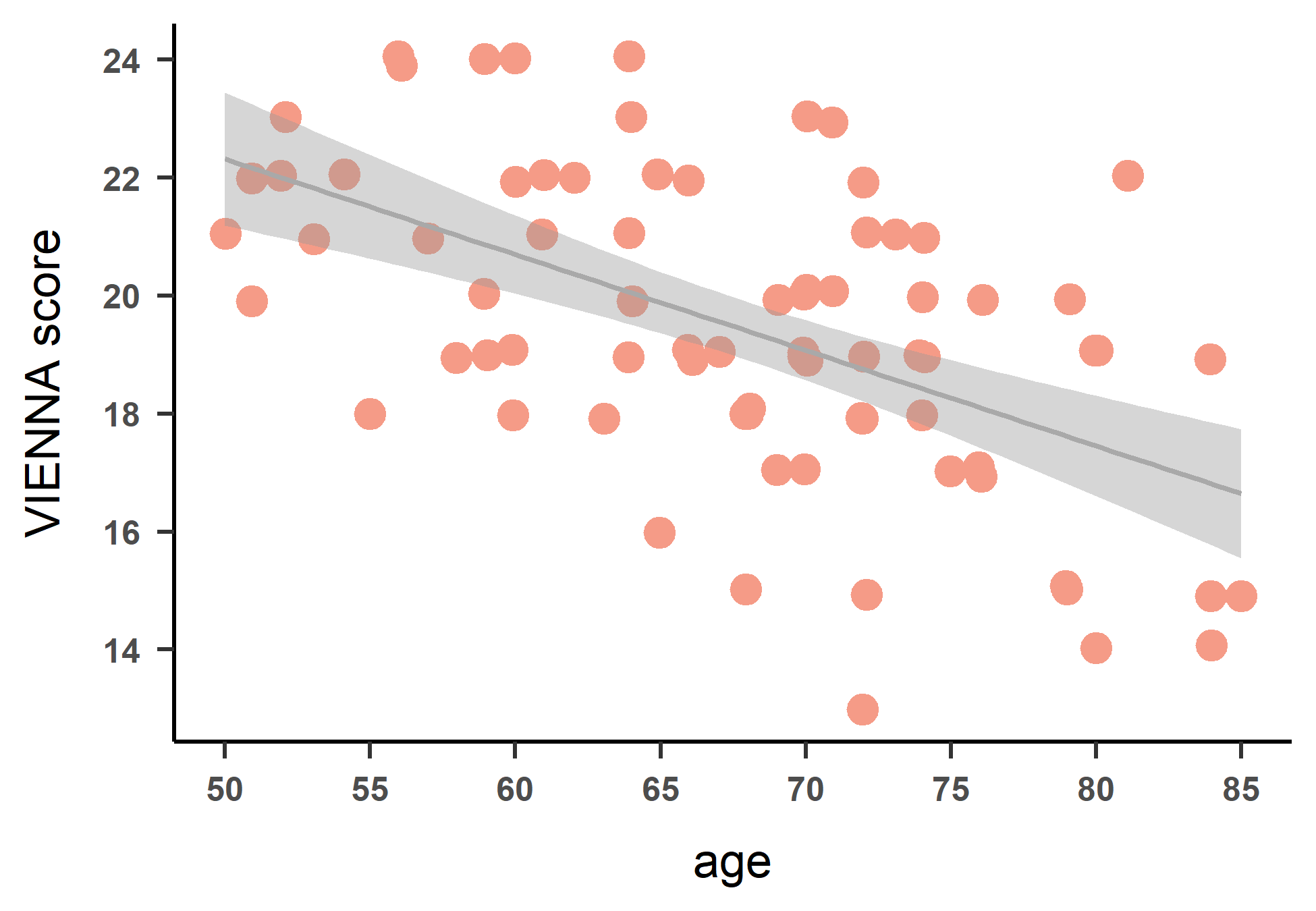

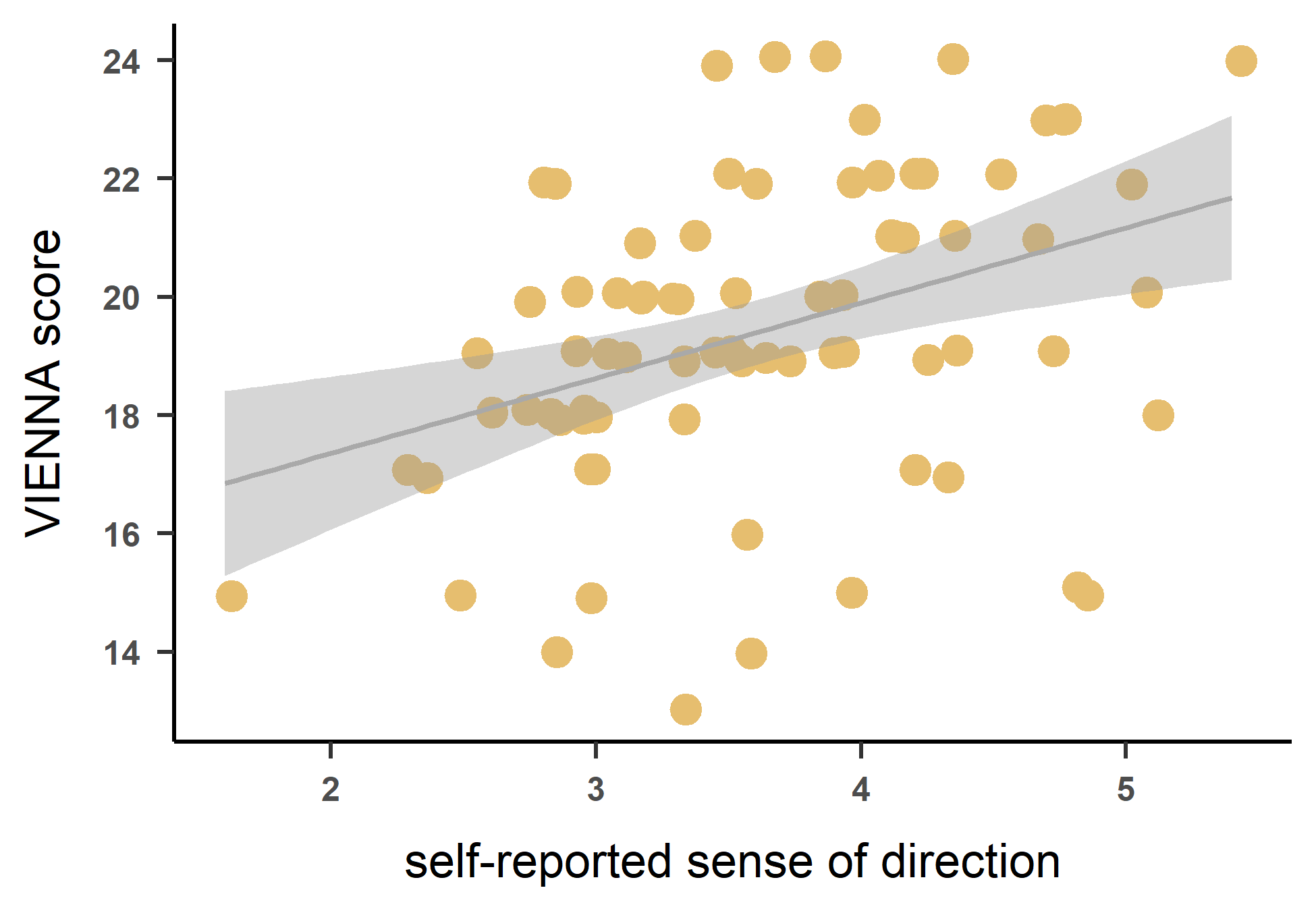


**Figure C1.** Scatterplots of significant correlations between VIENNA performance and demographic variables and questionnaire data.


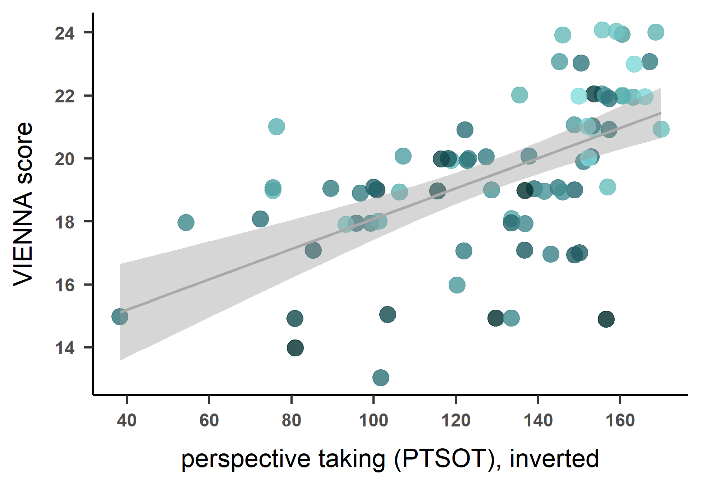

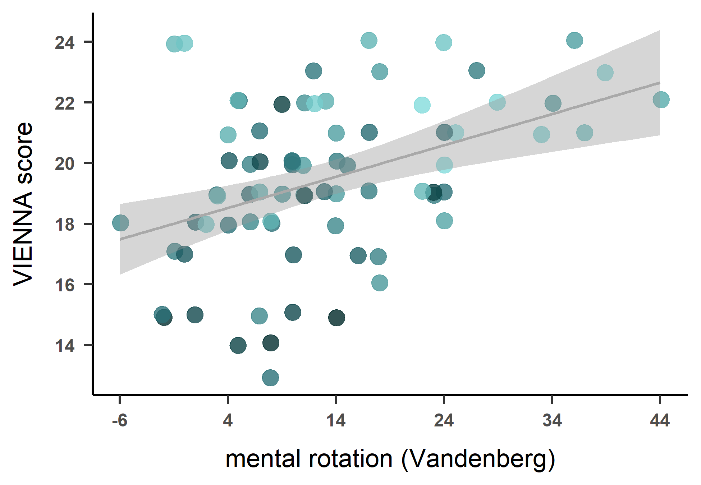

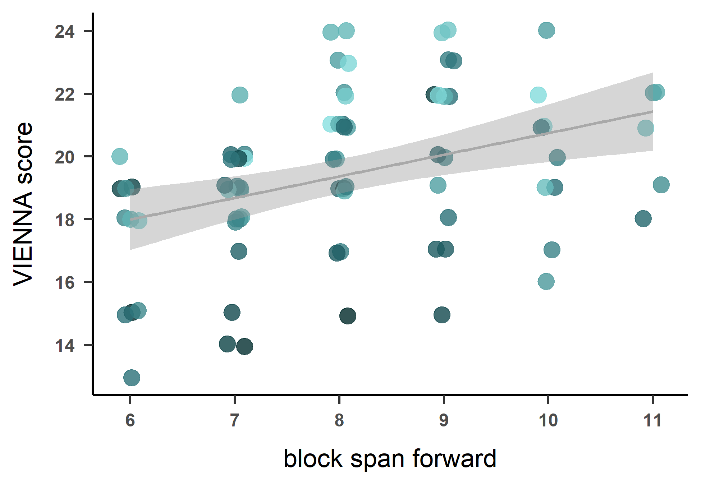

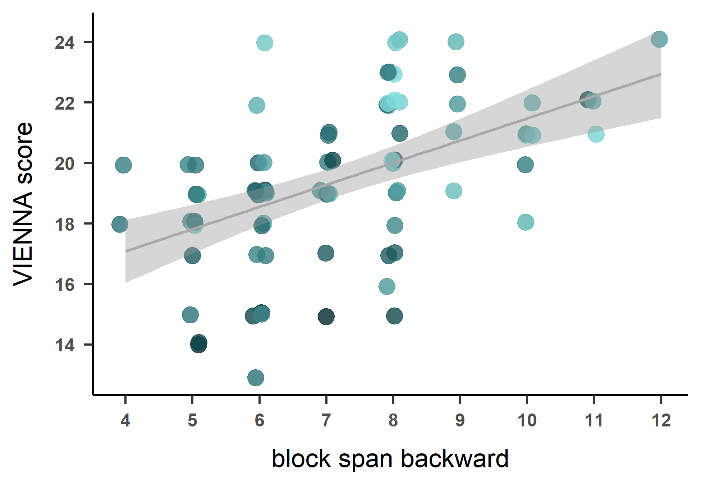

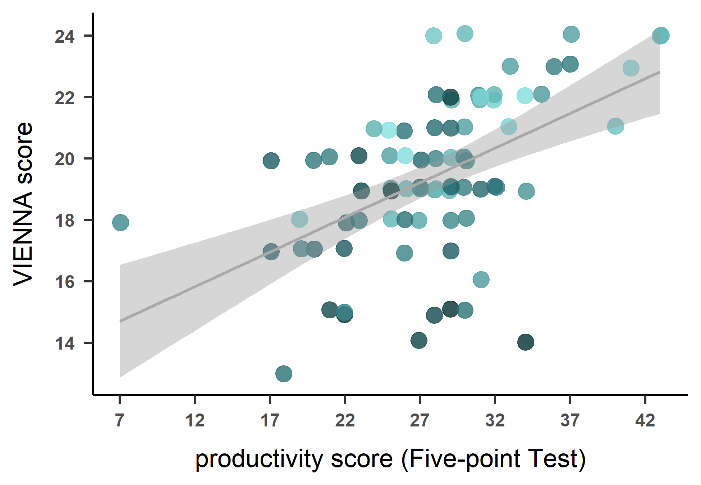

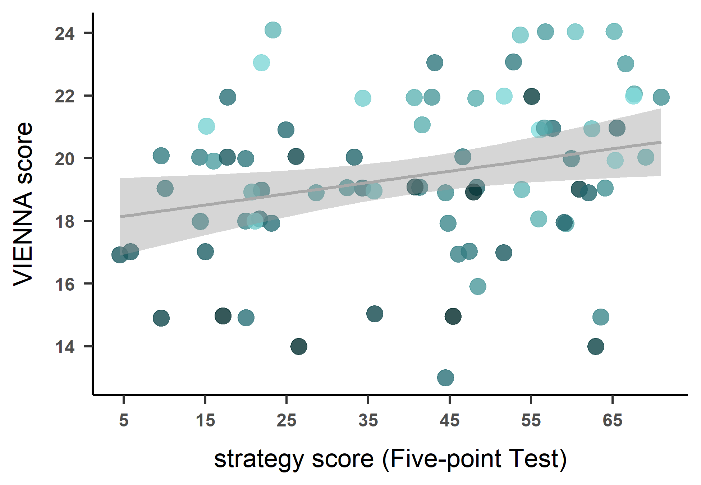

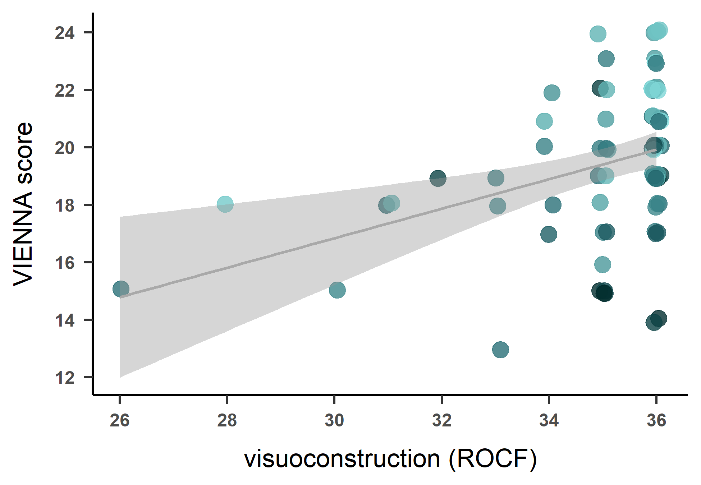

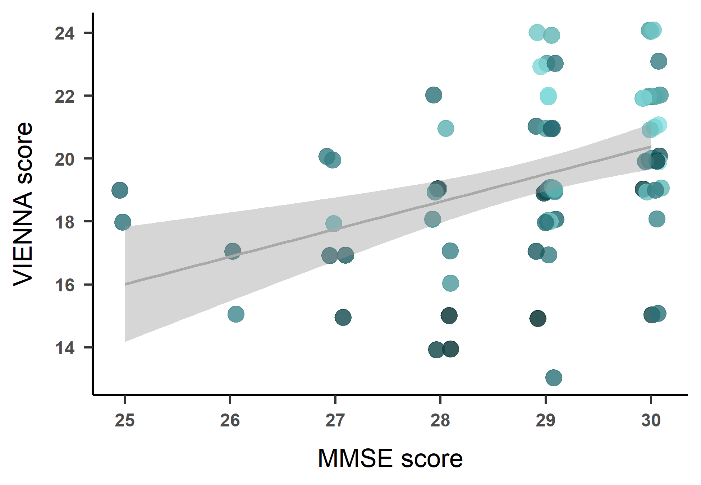
**Figure C2.** Scatterplots of significant correlations of cognitive tests with VIENNA performance. Cognitive variables are color coded for age from 50 (light bluegreen) to 85 (dark bluegreen).


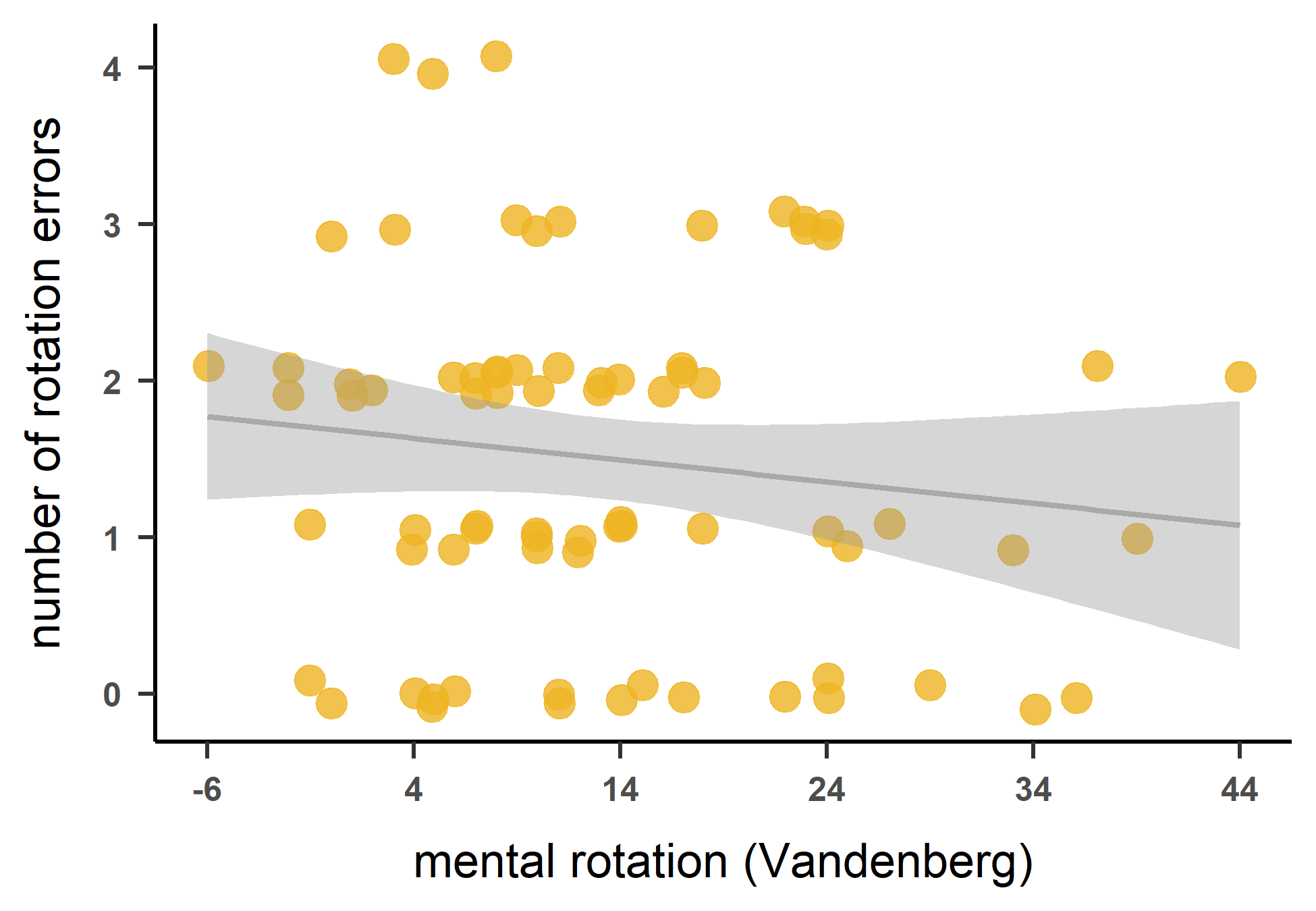

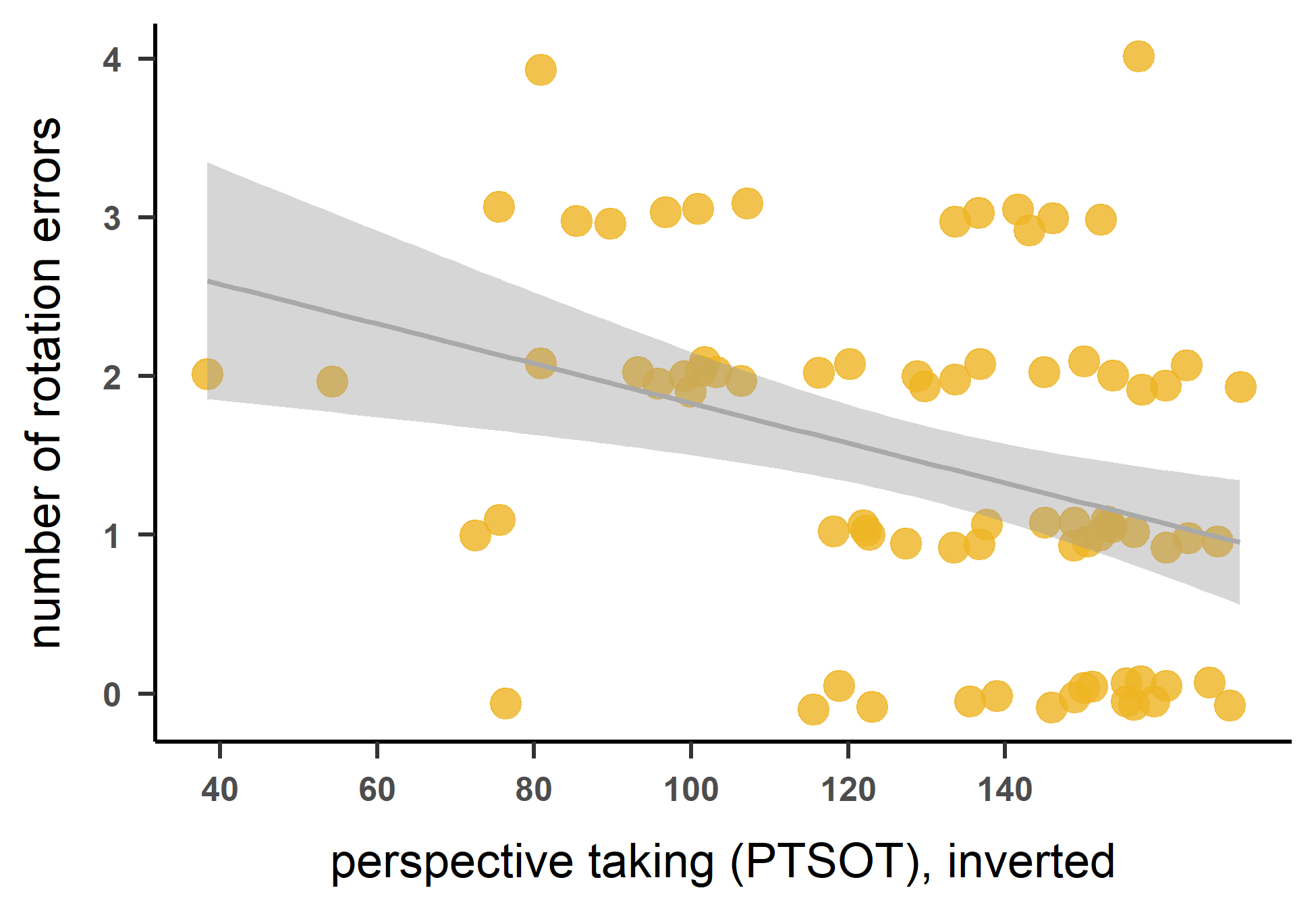


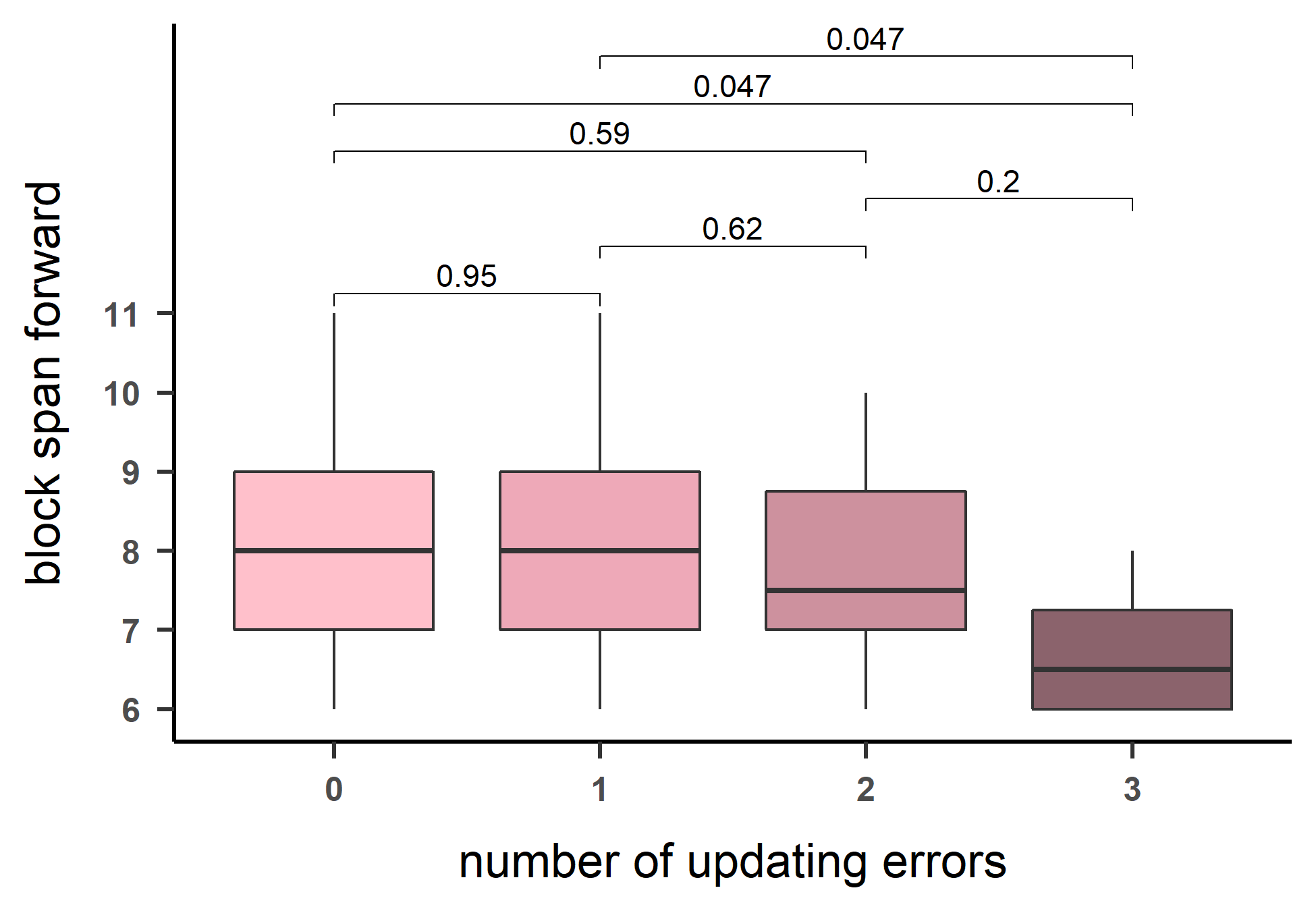

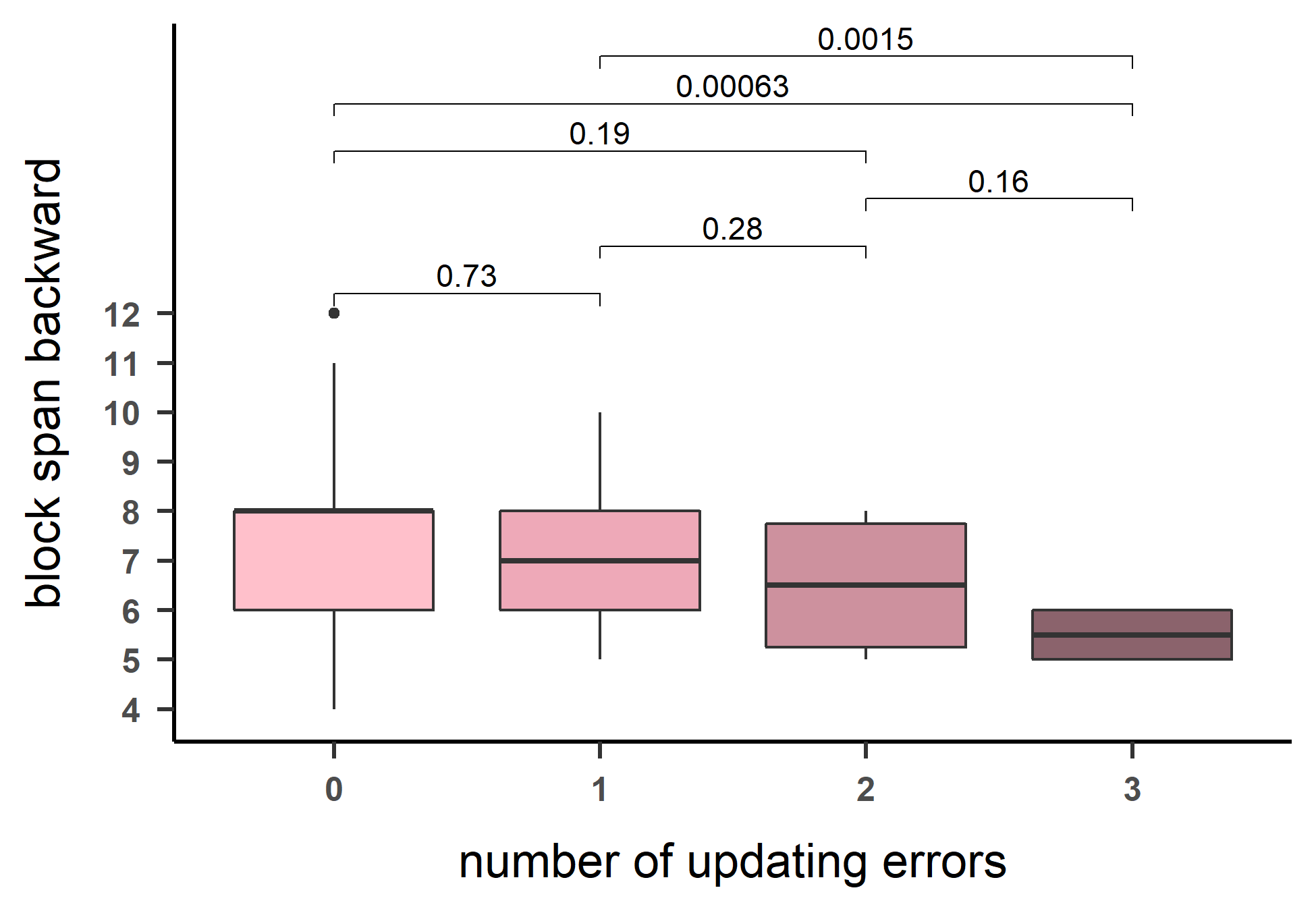


**Figure C3.** Visualization of the relationship VIENNA error types rotation with mental rotation and perspective taking and updating with short-term and working memory.

**Table C1.** Multiple regression model 1, explaining 59 percent of the variance in the VIENNA performance.

| Predictor | $b$ | 95% CI | $t$(64) | $p$ |
| --- | --- | --- | --- | --- |
| Intercept | 19.50 | [19.08, 19.92] | 93.34 | < .001 |
| Age | -0.92 | [-1.41, -0.44] | -3.80 | < .001 |
| FSBSOD | 0.35 | [-0.15, 0.85] | 1.41 | .164 |
| ROCF copy | 0.47 | [0.00, 0.95] | 1.98 | .052 |
| Block span forward | 0.19 | [-0.37, 0.74] | 0.67 | .507 |
| Block span backward | 0.30 | [-0.24, 0.84] | 1.12 | .268 |
| Vandenberg MRT | -0.23 | [-0.78, 0.31] | -0.86 | .393 |
| PTSOT | 0.53 | [-0.03, 1.08] | 1.88 | .064 |
| FPT productivity | 0.53 | [0.02, 1.03] | 2.07 | .042 |
| FPT strategy | -0.03 | [-0.52, 0.46] | -0.13 | .901 |

*Note.* FSBSOD = Freiburg Santa Barbara Sense of Direction Scale, ROCF = Rey-Osterrieth Complex Figure Test, MRT = Mental Rotation Test, PTSOT = Perspective Taking Test, FPT = Five-point Test.
